# Quantitative prediction of rate constants and its application to organic emitters

**DOI:** 10.1038/s41467-024-49069-4

**Published:** 2024-06-03

**Authors:** Katsuyuki Shizu, Hironori Kaji

**Affiliations:** Institute for Chemical Research, Kyoto University, Uji, Kyoto 611-0011 Japan

**Keywords:** Materials for devices, Materials chemistry

## Abstract

Many phenomena in nature consist of multiple elementary processes. If we can predict all the rate constants of respective processes quantitatively, we can comprehensively predict and understand various phenomena. Here, we report that it is possible to quantitatively predict all related rate constants and quantum yields without conducting experiments, using multiple-resonance thermally activated delayed fluorescence (MR–TADF) as an example. MR–TADFs are excellent emitters because of its narrow emission, high luminescence efficiency, and chemical stability, but they have one drawback: slow reverse intersystem crossing (RISC), leading to efficiency roll-off and reduced device lifetime. Here, we show a quantum chemical calculation method for quantitatively obtaining all the rate constants and quantum yields. This study reveals a strategy to improve RISC without compromising other important factors: radiative decay rate constants, photoluminescence quantum yields, and emission linewidths. Our method can be applied in a wide range of research fields, providing comprehensive understanding of the mechanism including the time evolution of excitons.

## Abstract (web summary)

The quantitative prediction of rate constants and quantum yields is crucial to understand various phenomena and their mechanisms in nature. Here, the authors apply quantum chemical calculation to reproduce experimental data of multiple-resonance thermally activated delayed fluorescence emitters.

## Introduction

Multiple-resonance thermally activated delayed fluorescence (MR–TADF) has attracted substantial attention in organic light-emitting diodes (OLEDs) research because it can realise high external quantum efficiency and narrow electroluminescence spectra with high colour purity^1–3^. Hatakeyama et al. reported the first MR–TADF molecule (DABNA-1) in 2016^1^ by incorporating B and N atoms into a carbon-based molecular structure. In DABNA-1, the highest occupied molecular orbital (HOMO) and the lowest unoccupied molecular orbital (LUMO) distributions are spatially separated on different atoms, resulting in a relatively small energy difference Δ*E*(T_1_ → S_1_) of 0.15–0.2 eV between the lowest excited singlet state (S_1_) and lowest triplet state (T_1_). The Δ*E*(T_1_ → S_1_) value is sufficiently small to cause TADF but rather large; the rate constant (*k*_RISC_) for reverse intersystem crossing (RISC) is small (9.9 × 10^3^ s^−1^)^1^. Since the report of DABNA-1, a number of MR–TADF molecules have been developed; however, most of them exhibit *k*_RISC_ values on the order of 10^4^–10^5^ s^−1^ ^1–7^. The slow RISC is considered to cause triplet-related annihilation^8^, resulting in efficiency roll-off in OLEDs. The slow RISC also reduces the device lifetime of OLEDs. Thus, there is an urgent need to develop MR–TADF emitters with large *k*_RISC_ to solve these problems without sacrificing the rate constant of fluorescence from S_1_ to the ground state (S_0_) (*k*_F_(S_1_ → S_0_) or simply *k*_F_), the photoluminescence quantum yield (PLQY), and the colour purity. Recently, Yasuda’s group developed MR–TADF materials with *k*_RISC_ of 10^8^ s^−1^ by utilising the heavy atom effect of selenium (Se) atoms^9^. However, the *k*_F_ (~10^5^ s^−1^) was more than two orders of magnitude smaller than the oxygen (O) type analogue. Yang’s group developed Se-containing MR–TADF materials. The *k*_RISC_ of 10^6^ s^−1^ and the *k*_F_ of ~10^7^ s^−1^ are more well-balanced^10^. However, the efficiency roll-off problem has not been well solved; simultaneous realisation of *k*_RISC_ > 10^7^ s^−1^ and *k*_F_ > 10^7^ s^−1^ is desirable.

Among all the reported MR–TADF molecules, the RISC mechanisms have been analysed in detail for DABNA-1, DABNA-2, and ν-DABNA. Quantum chemical calculations^11–14^ and time-resolved photoluminescence (PL) measurements^15^ indicate that the total RISC process of the three materials occurs via higher triplet states (T_*n*_, *n* ≥ 2), typically T_2_ (Kim et al.^13^ carried out an analysis including from T_1_ to T_3_), although only the direct T_1_ → S_1_ RISC has initially been considered. When RISC occurs via T_2_ (T_1_ → T_2_ → S_1_), *k*_RISC_ increases with decreasing T_2_ → S_1_ (especially when S_1_ is higher in energy than T_2_) and T_1_ → T_2_ energy gaps (denoted as Δ*E*(T_2_ → S_1_) and Δ*E*(T_1_ → T_2_), respectively), and increasing S_1_–T_2_ spin–orbit coupling (SOC(S_1_–T_2_)). A small |Δ*E*(T_2_ → S_1_)| and large SOC(S_1_–T_2_) accelerate the T_2_ → S_1_ transition, and a small Δ*E*(T_1_ → T_2_) accelerates the T_1_ → T_2_ internal up-conversion.

We have reported three methods to enhance RISC: (1) intervening in a locally excited state between charge transfer type singlet (^1^CT) and triplet (^3^CT) states^16^, (2) using the fluctuational effect for ^3^CT → ^1^CT RISC^17^, although it seemingly violates the El-Sayed rule^18^, and (3) using the heavy atom effect^19–21^. Regarding the third method, a practical strategy for increasing *k*_RISC_ is assumed to incorporate third-, fourth-, or lower-row elements to enhance SOC by their heavy atom effect. Various O-, sulfur- (S-), and Se-containing molecules have been developed for conventional TADF emitters composed of donor and acceptor segments^10,22–30^; *k*_RISC_ is enhanced by 2–20 times via O → S substitution^10,22,23,25^, and up to 50 times via O → Se substitution^10^. The carbonyl (C = O) group can also enhance SOC because of the n–π* orbital; as evidenced by benzophenone, a representative carbonyl compound, having a large rate constant (*k*_ISC_) for intersystem crossing (ISC) of ~10^11^ s^−1^ ^31^. The C = O group is a promising alternative to S and Se for enhancing SOC with only C, H, and O atoms^5,6,32–35^. A C = O-containing MR–TADF emitter developed by the Zysman–Colman group (DDiKTa^6^) exhibited a larger *k*_RISC_ of 6.3 × 10^5^ s^−1^ than that of ν-DABNA (*k*_RISC_ = 2.0 × 10^5^ s^−1^), although DDiKTa had a larger Δ*E*(T_1_ → S_1_) of 0.16 eV than ν-DABNA (0.07 eV), suggesting that the C = O groups in DDiKTa accelerated RISC.

Thus, understanding the excited-state decay mechanisms of MR-TADF emitters is important to design novel materials with enhanced TADF properties. To understand the decay mechanisms of a TADF emitter, it is common to determine the rate constants of electronic transitions from the experimental PLQY and transient photoluminescence decay curve fitted by a linear combination of exponential decay functions. A comprehensive understanding of the emission mechanism is achieved only when the rate constants for all elementary electronic transitions have been determined. However, it is difficult to determine all rate constants when the number of the rate constants is larger than that of experimentally determined fitting parameters (specifically, when singlet and triplet states energetically higher than S_1_ and T_1_ are involved). Our theoretical method allows us to quantitatively predict rate constants and quantum yields, including those inaccessible from experiments. Our method also clarifies the quantitative dynamics (time evolutions) of excitons, providing a comprehensive understanding of the TADF mechanism. Therefore, our method proposed in this study offers a guideline for designing TADF emitters with enhanced properties.

**Fig. 1:**
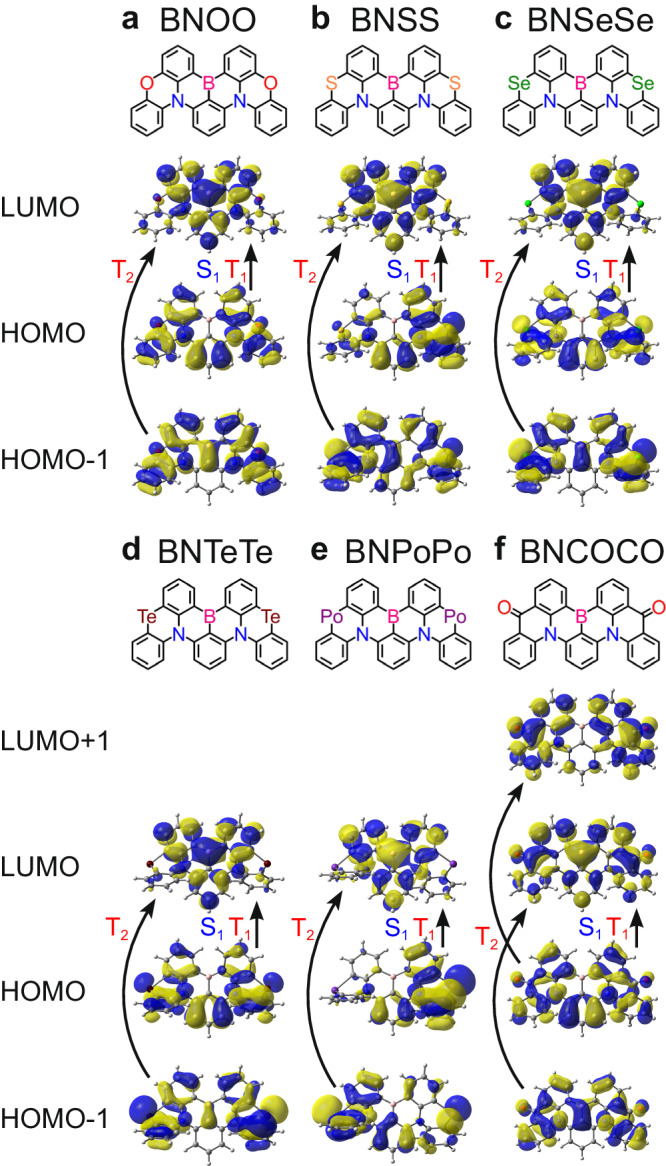
Molecular structures and HOMO−1, HOMO, and LUMO distributions. **a** BNOO, **b** BNSS, **c** BNSeSe, **d** BNTeTe, **e** BNPoPo, and **f** BNCOCO. HOMO and LUMO are the highest occupied molecular orbital and lowest unoccupied molecular orbital, respectively.

Here, we report a quantitative theoretical investigation of the emission mechanism of MR–TADF molecules, focusing on the acceleration of RISC. As with various phenomena, the emission here consists of multiple elementary processes. The quantitative description of the rate constants not only for RISC but also for all relevant elementary processes is of primary importance because such a description enables a comprehensive understanding and prediction of the emission mechanism. We investigate the RISC mechanism of previously synthesised MR–TADF emitters (BNOO, BNSS, and BNSeSe) (Fig. 1), reported by Yang’s group^10^. Here, we define a total ISC/RISC rate constant (*k*_toISC_/*k*_toRISC_) as the entire ISC/RISC process involving both the S_1_ → T_1_/T_1_ → S_1_ and S_1_ → T_2_ ↔ T_1_/T_1_ ↔ T_2_ → S_1_ transitions, which correspond to the experimentally obtained values (see the “Methods” section). Electronic states higher than S_1_ and T_2_ are not required to be considered because they are energetically well separated. All the calculated *k*_toISC_ (8.6 × 10^7^, 2.0 × 10^8^, and 1.0 × 10^9^ s^−1^ for BNOO, BNSS, and BNSeSe, respectively) and *k*_toRISC_ (9.2 × 10^3^, 2.5 × 10^5^, and 1.5 × 10^6^ s^−1^, respectively) in this study well-reproduce the experimental *k*_toISC_ (7.5 × 10^7^, 1.5 × 10^8^, and 4.9 × 10^8^ s^−1^, respectively) and *k*_toRISC_ (4.3 × 10^4^, 1.9 × 10^5^, and 2.0 × 10^6^ s^−1^, respectively). The calculations also clarify how the S and Se atoms accelerate RISC. All the calculated values of Δ*E*(T_1_ → S_1_), *k*_F_(S_1_ → S_0_), and PLQY (*Φ*) as well as the prompt and TADF contributions, also reasonably reproduce the experimental results (Table 1); indicating the validity of our calculation method. The calculated *k*_toRISC_ of BNOO slightly deviates from the experimental value because of the overestimation of Δ*E*(T_1_ → S_1_) (when we use the experimental Δ*E*(T_1_ → S_1_) of 0.15 eV instead of the calculated value of 0.21 eV, *k*_toRISC_ is calculated to be 8.2 × 10^4^ s^−1^, close to the experimental value of 4.3 × 10^4^ s^−1^).

**Table 1:** Calculated energies, energy gaps, rate constants, quantum yields, spin-orbit couplings, and emission linewidths of BNOO, BNSS, BNSeSe, BNTeTe, BNPoPo, and BNCOCO

	BNOO	BNSS	BNSeSe	BNTeTe	BNPoPo	BNCOCO
*E*(S_1_) (eV)	2.51 (2.54)	2.44 (2.55)	2.51 (2.58)	2.52	2.41	2.55
*E*(T_1_) (eV)	2.30 (2.39)	2.30 (2.42)	2.37 (2.44)	2.38	2.26	2.41
Δ*E*(T_1_ → S_1_) (eV)	0.21 (0.15)	0.14 (0.13)	0.14 (0.14)	0.14	0.14	0.14
Δ*E*(T_2_ → S_1_) (eV)	0.08	−0.04	−0.05	−0.06	−0.12	0.03
Δ*E*(T_1_ → T_2_) (eV)	0.13	0.18	0.19	0.20	0.26	0.11
*k*_toISC_ (s^−1^)	8.6 × 10^7^ (7.5 × 10^7^)	2.0 × 10^8^ (1.5 × 10^8^)	1.0 × 10^9^ (0.5 × 10^9^)	6.6 × 10^9^	4.3 × 10^10^	6.5 × 10^8^
*k*_toRISC_(T_1_) (s^−1^)	8.4	2.0 × 10^3^	1.2 × 10^5^	3.0 × 10^6^	4.9 × 10^7^	39
*k*_toRISC_(T_2_) (s^−1^)	9.2 × 10^3^	2.5 × 10^5^	1.4 × 10^6^	7.2 × 10^6^	6.9 × 10^6^	9.9 × 10^5^
*k*_toRISC_ (s^−1^)	9.2 × 10^3^ (4.3 × 10^4^)	2.5 × 10^5^ (1.9 × 10^5^)	1.5 × 10^6^ (2.0 × 10^6^)	1.0 × 10^7^	5.6 × 10^7^	9.9 × 10^5^
*k*_toRISC_′ (s^−1^)	9.3 × 10^3^	2.5 × 10^5^	1.5 × 10^6^	1.0 × 10^7^	n. d.	9.9 × 10^5^
*k*_F_(S_1_ → S_0_) (s^−1^)	2.7 × 10^8^ (8.2 × 10^7^)	2.2 × 10^8^ (4.5 × 10^7^)	2.5 × 10^8^ (2.6 × 10^7^)	2.4 × 10^8^	1.5 × 10^8^	4.7 × 10^8^
*k*_NR_(S_1_ → S_0_) (s^−1^)	2.0 × 10^7^	2.1 × 10^7^	1.8 × 10^7^	1.8 × 10^7^	2.0 × 10^7^	1.2 × 10^7^
*k*_ISC_(S_1_ → T_1_) (s^−1^)	7.9 × 10^4^	1.6 × 10^6^	8.7 × 10^7^	1.9 × 10^9^	3.7 × 10^10^	2.6 × 10^4^
*k*_ISC_(S_1_ → T_2_) (s^−1^)	8.6 × 10^7^	2.0 × 10^8^	9.4 × 10^8^	4.7 × 10^9^	5.3 × 10^9^	6.5 × 10^8^
*k*_IC_(T_2_ → T_1_) (s^−1^)	9.9 × 10^12^	2.4 × 10^12^	2.2 × 10^12^	1.4 × 10^12^	4.2 × 10^11^	4.7 × 10^12^
*k*_RISC_(T_2_ → S_1_) (s^−1^)	1.3 × 10^6^	2.6 × 10^8^	1.8 × 10^9^	1.5 × 10^10^	1.8 × 10^11^	7.8 × 10^7^
*k*_NR_(T_2_ → S_0_) (s^−1^)	0.68	2.7	1.9 × 10^2^	2.1 × 10^3^	2.9 × 10^4^	0.66
*k*_Phos_(T_2_ → S_0_) (s^−1^)	92	1.5 × 10^3^	9.6 × 10^3^	5.7 × 10^4^	3.4 × 10^5^	3.4 × 10^3^
*k*_IC_(T_1_ → T_2_) (s^−1^)	7.1 × 10^10^	2.4 × 10^9^	1.7 × 10^9^	6.5 × 10^8^	1.6 × 10^7^	6.1 × 10^10^
*k*_RISC_(T_1_ → S_1_) (s^−1^)	8.5	2.0 × 10^3^	1.2 × 10^5^	3.0 × 10^6^	4.9 × 10^7^	40
*k*_NR_(T_1_ → S_0_) (s^−1^)	3.8	14	1.4 × 10^2^	1.1 × 10^3^	3.5 × 10^4^	0.44
*k*_Phos_(T_1_ → S_0_) (s^−1^)	1.6	10	4.9 × 10^2^	6.6 × 10^3^	9.7 × 10^4^	0.25
*k*_Prompt_ (s^−1^)	3.7 × 10^8^ (1.9 × 10^8^)	4.4 × 10^8^ (2.0 × 10^8^)	1.3 × 10^9^ (0.5 × 10^9^)	6.8 × 10^9^	4.1 × 10^10^	1.1 × 10^9^
*k*_Delayed_ (s^−1^)	7.1 × 10^3^ (26 × 10^3^)	1.4 × 10^5^ (0.5 × 10^5^)	3.1 × 10^5^ (1.0 × 10^5^)	3.9 × 10^5^	3.5 × 10^5^	4.2 × 10^5^
*k*_TADF_ = *k*_toR_(S_1_) (s^−1^)	6.6 × 10^3^	1.3 × 10^5^	2.9 × 10^5^	3.6 × 10^5^	1.9 × 10^5^	4.1 × 10^5^
*k*_toR_(T_1_) (s^−1^)	1.6	10	4.9 × 10^2^	6.6 × 10^3^	9.7 × 10^4^	0.25
*k*_toR_(T_2_) (s^−1^)	0.65	1.4	7.1	28	13	43
*k*_toR_ (s^−1^)	6.6 × 10^3^	1.3 × 10^5^	2.9 × 10^5^	3.7 × 10^5^	2.9 × 10^5^	4.1 × 10^5^
*Φ*	0.93 (0.71)	0.91 (0.91)	0.93 (1.0)	0.93	0.83	0.97
*Φ*_NR_(S_1_)	0.07	0.09	0.07	0.07	0.07	0.03
*Φ*_NR_(T_1_)	0.00	0.00	0.00	0.00	0.10	0.00
*Φ*_NR_(T_2_)	0.00	0.00	0.00	0.00	0.00	0.00
*Φ*_Prompt_	0.71 (0.43)	0.50 (0.23)	0.19 (0.05)	0.04	0.003	0.41
*Φ*_TADF_	0.22 (0.28)	0.41 (0.68)	0.74 (0.95)	0.87	0.55	0.56
*Φ*_Phos_(T_1_)	0.00	0.00	0.00	0.02	0.28	0.00
*Φ*_Phos_(T_2_)	0.00	0.00	0.00	0.00	0.00	0.00
S_0_-T_1_ SOC (cm^−1^)	2.12	4.07	13.5	37.7	202	0.76
S_0_-T_2_ SOC (cm^−1^)	0.96	1.92	16.9	56.5	204	0.97
S_1_-T_1_ SOC (cm^−1^)	0.04	0.12	0.84	3.92	17.7	0.01
S_1_-T_2_ SOC (cm^−1^)	0.55	1.17	3.32	10.6	59.1	1.01
FWHM (nm)	50–53 (51)	50–53 (53)	47–50 (47)	47–50	52–56	43–46

Some values are time-dependent; the values here are those at the equilibrium states, which correspond to the experimentally observable ones. The values in the parentheses are experimental data reported by Hu et al.^10^. *k*_TADF_ for BNOO/BNSS/BNSeSe/BNTeTe/BNPoPo/BNCOCO is defined in the time domain longer than 100/100/10/ 10/1/10 ns. *k*_toRISC_ for BNOO/BNSS/BNSeSe/BNTeTe/BNPoPo/BNCOCO is defined in the time domain longer than 1/10/10/10/1/10 ns (Supplementary Figs. 2 and 3). *k*_toRISC_′ is the total RISC rate constant calculated by our previously proposed method^20^. n.d. means not determined. *k*_toR_(S_1_), *k*_toR_(T_1_), and *k*_toR_(T_2_) are the contributions from S_1_ → S_0_ fluorescence, T_1_ → S_0_ phosphorescence, and T_2_ → S_0_ phosphorescence to *k*_toR_, respectively: *k*_toR_ = *k*_toR_(S_1_) + *k*_toR_(T_1_) + *k*_toR_(T_2_); *k*_toR_(S_1_) = *k*_F_(S_1_ → S_0_) × [S_1_]/([S_1_] + [T_1_] + [T_2_]); *k*_toR_(T_1_) = *k*_Phos_(T_1_ → S_0_) × [T_1_]/([S_1_] + [T_1_] +[T_2_]); *k*_toR_(T_2_) = *k*_Phos_(T_2_ → S_0_) × [T_2_]/([S_1_] + [T_1_] + [T_2_]). *k*_toRISC_(T_1_) and *k*_toRISC_(T_2_) are the contributions from T_1_ → S_1_ and T_2_ → S_1_ ISCs to *k*_toRISC_, respectively. *k*_toRISC_ = *k*_toRISC_(T_1_) + *k*_toRISC_(T_2_); *k*_toRISC_(T_1_) = *k*_RISC_(T_1_ → S_1_) × [T_1_]/([T_1_] + [T_2_]); *k*_toRISC_(T_2_) = *k*_RISC_(T_2_ → S_1_) × [T_2_]/([T_1_] + [T_2_]).

Next, we discuss the impacts of further heavy atom effects on RISC and the possibility of further increasing *k*_toRISC_ by using tellurium (Te), polonium (Po), and C = O substitutions (Fig. 1). Our calculations predict that a Te-containing emitter (BNTeTe) exhibits one order of magnitude larger *k*_toRISC_ of 10^7^ s^−1^ than BNSeSe (*k*_toRISC_ ~ 10^6^ s^−1^). Although Po is highly radioactive, we theoretically investigate a Po-containing emitter (BNPoPo) to understand the heavy atom effect. Our calculations indicate that BNPoPo exhibits both TADF and phosphorescence. We calculate the *k*_toRISC_ of a C = O-containing molecule (BNCOCO) to be 10^6^ s^−1^. Compared with Se, the C = O group has a smaller SOC-enhancement ability but provides a sufficient T_1_ → T_2_ internal up-conversion, resulting in *k*_toRISC_ that is comparable to that of BNSeSe. The calculated *k*_F_(S_1_ → S_0_)s are the same order of magnitude (10^8^ s^−1^) for all the compounds investigated in this study. Their calculated PLQYs remain high, indicating that we can enhance RISC without sacrificing radiative decays nor PLQYs, which is otherwise a trade-off in TADF emitters. Finally, we theoretically predict the linewidths (full width at half maximum (FWHM)) of the PL spectra for these molecules. The calculated FWHM values are almost identical for BNOO, BNSS, and BNSeSe; agreeing well with the experimental values. Those for designed BNTeTe, BNPoPo, and BNCOCO are comparable to those for BNOO, BNSS, and BNSeSe; indicating that the acceleration of *k*_RISC_ in this study also does not sacrifice the FWHM values.

## Results

### Computational results for BNOO, BNSS, and BNSeSe

**Fig. 2:**
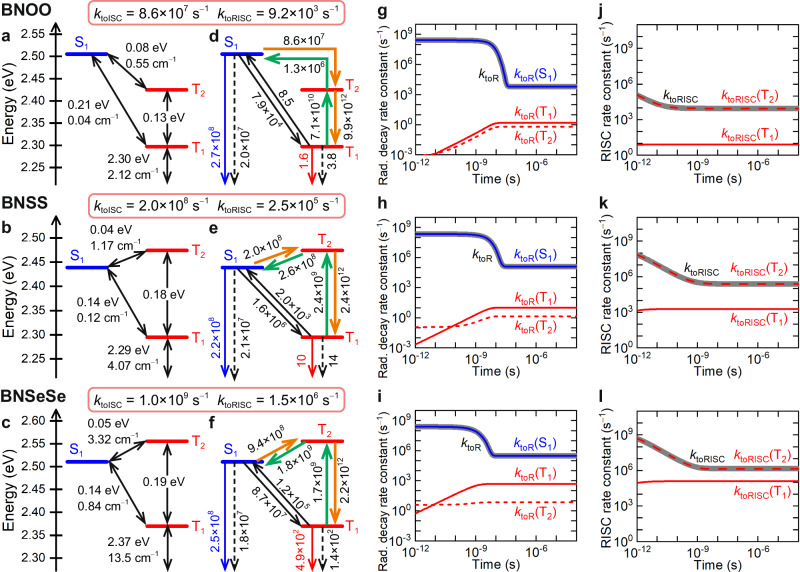
Excited-state decay mechanism. **a**–**c** Calculated excited-state energy diagram, energy differences (eV), and spin–orbit couplings (cm^−1^) and **d**–**f** rate constants (s^−1^) for BNOO, BNSS, and BNSeSe. **g**–**i** Calculated *k*_toR_, *k*_toR_(S_1_), *k*_toR_(T_1_), and *k*_toR_(T_2_) for BNOO, BNSS, and BNSeSe. **j**–**l** Calculated *k*_toRISC_, *k*_toRISC_(T_1_), and *k*_toRISC_(T_2_) for BNOO, BNSS, and BNSeSe. In **d**–**f**, the solid orange arrows depict the dominant S_1_ → T_1_ pathway, the solid green arrows depict the dominant T_1_ → S_1_ pathway, the solid black arrows depict the minor S_1_ → T_1_ and T_1_ → S_1_ pathways, the solid blue arrows depict the S_1_ → S_0_ fluorescence, the solid red arrows depict the T_1_ → S_0_ phosphorescence, and the dashed arrows depict S_1_ → S_0_ and T_1_ → S_0_ nonradiative decays. In **g**–**i**, the solid grey curves depict *k*_toR_, the blue curves depict *k*_toR_(S_1_), the solid red curves depict *k*_toR_(T_1_), and the dashed red curves depict *k*_toR_(T_2_). In **j**–**l**, the solid grey curves depict *k*_toRISC_, the solid red curves depict *k*_toRISC_(T_1_), and the dashed red curves depict *k*_toRISC_(T_2_). Source data for figure g-l are provided as a Source Data file.

Our theoretical analysis is based on rate constant calculations. Details of the method of calculating the rate constants for fluorescence from S_1_ to S_0_ (*k*_F_(S_1_ → S_0_)), T_*n*_ → S_0_ phosphorescence (*k*_Phos_(T_*n*_ → S_0_)), S_1_ → S_0_ nonradiative decay (*k*_NR_(S_1_ → S_0_)), T_*n*_ → S_0_ nonradiative decay (*k*_NR_(T_*n*_ → S_0_)), T_*m*_ → T_*n*_ internal conversion (*k*_IC_(T_*m*_ → T_*n*_)), S_1_ → T_*n*_ ISC (*k*_ISC_(S_1_ → T_*n*_)), and T_*n*_ → S_1_ RISC (*k*_RISC_(T_*n*_ → S_1_)) (*m*, *n* = 1, 2, *m* ≠ *n*) are described in the “Methods” section. As is well known, conventional time-dependent density functional theory (TD-DFT) methods substantially overestimate Δ*E*(T_1_ → S_1_) of MR–TADF emitters, which can be solved by calculations including double-excitation configurations^36^. Several wave-function-based methods (including the second-order algebraic-diagrammatic construction ADC(2) and the spin-component-scaling second-order approximate coupled-cluster (SCS–CC2)) give more reliable Δ*E*(T_1_ → S_1_) than TD–DFT methods^36^. Recently, it has become common to use different theoretical methods for different molecular properties^13,37,38^. For example, Lin et al. calculated excitation energies with the TD-B3LYP/6-31G(d) method, and then, the T_1_ energy was corrected using Δ*E*(T_1_ → S_1_) obtained from the SCS–CC2/def2-TZVP calculation^38^. Tamm–Dancoff approximation (TDA)–DFT methods with double hybrid density functionals such as TDA–B2-PLYP are emerging alternative approaches for considering double-excitation configurations^39^. TDA–DFT methods have the advantage of low computational cost compared with ADC(2) and SCS–CC2. Here, we compared the Δ*E*(T_1_ → S_1_) values calculated with the three methods as well as that by the conventional TD–DFT (B3LYP) method (blue texts in Supplementary Tables 13 and 14). Supplementary Table 13 also shows the experimental values. The TDA–B2-PLYP method provided the Δ*E*(T_1_ → S_1_) values closest to the experimental results for BNSS and BNSeSe. Therefore, we used the TDA–B2-PLYP method (TDA–DFT with the B2-PLYP double-hybrid functional and def2-TZVP basis set) for calculating Δ*E*(T_1_ → S_1_), which we combined with the energy levels of S_1_ and T_2_ calculated by the TD–DFT with the B3LYP functional and 6-31 G(d)+SDD basis set (TD–B3LYP method). All the other calculations (SOCs, vibronic coupling constants, transition dipole moments, and permanent dipole moments) were carried out by the TD–B3LYP method (see Methods section and Supplementary Method 2 for the details). We performed the TDA–B2-PLYP calculations with the ORCA 5.0.3 program package (FACCTs, Cologne, Germany)^40–42^ and the ADC(2) and SCS–CC2 calculations with the TURBOMOLE program package^43^. Table 1 shows the calculated and experimental data. Figure 2a–f shows the calculated excited-state energy diagrams, energy gaps, SOCs, and rate constants for BNOO, BNSS, and BNSeSe. Figure 2g–l shows the time evolutions of respective rate constants. The calculated Δ*E*(T_1_ → S_1_) for BNSS and BNSeSe agree with the experimental values (calculated and experimental Δ*E*(T_1_ → S_1_) are 0.14 and 0.13 eV, respectively, for BNSS and they are both 0.14 eV for BNSeSe), although we found a slight deviation by 0.06 eV for BNOO (the Δ*E*(T_2_ → S_1_) and Δ*E*(T_2_ → T_1_) values have not been determined experimentally). We can reasonably neglect the contributions of S_*n*_ (*n* ≥ 2) and T_*m*_ (*m* ≥ 3) as described above.

For BNOO, BNSS, and BNSeSe, S_1_ and T_2_ are energetically close (|Δ*E*(T_2_ → S_1_)| <0.1 eV), and the S_1_–T_2_ SOCs are stronger than the S_1_–T_1_ SOCs (Table 1 and Fig. 2a–c). As a result, *k*_ISC_(S_1_ → T_2_) is larger than *k*_ISC_(S_1_ → T_1_) by 1000 times for BNOO, 100 times for BNSS, and 10 times for BNSeSe. S_1_ and T_1_ of BNOO, BNSS, and BNSeSe are predominantly described as the HOMO–LUMO transitions, whilst their T_2_s are predominantly described as the HOMO−1-LUMO transitions (Fig. 1). The S_1_–T_2_ SOCs are more substantial than the S_1_–T_1_ SOCs because S_1_–T_2_ transitions are between different molecular orbital (MO) characters, whilst S_1_–T_1_ transitions are between similar MOs.

The larger S_1_–T_2_ SOCs compared with S_1_–T_1_ SOCs, as well as closer energy levels of S_1_–T_2_ compared with those of S_1_–T_1_, suggest faster transitions for T_2_-mediated ISC and RISC compared with those for direct S_1_–T_1_ ISC and RISC. The calculated *k*_toISC_ agrees well with the experimental values for BNOO, BNSS, and BNSeSe (Table 1). Regarding BNOO and BNSS, the stepwise S_1_ → T_2_ → T_1_ transition contributed to the entire ISC process and the direct S_1_ → T_1_ ISC was negligible (*k*_toISC_ ≈ *k*_ISC_(S_1_ → T_2_) ≫ *k*_ISC_(S_1_ → T_1_); *k*_IC_(T_2_ → T_1_) is very large). Regarding BNSeSe, although the contribution from the direct S_1_ → T_1_ ISC was not negligible, the stepwise S_1_ → T_2_ → T_1_ transition was still dominant (*k*_toISC_ ≈ *k*_ISC_(S_1_ → T_2_) and *k*_ISC_(S_1_ → T_1_) was one order smaller than *k*_ISC_(S_1_ → T_2_)). *k*_toISC_ increased with increasing atomic number for the chalcogen atoms (8.6 × 10^7^ s^−1^ < 2.0 × 10^8^ s^−1^ < 1.0 × 10^9^ s^−1^ for BNOO, BNSS, and BNSeSe, respectively), indicating that the O → S → Se substitution enhanced the S_1_–T_2_ SOC and accelerated the S_1_ → T_2_ ISC.

Next, we investigated RISC for BNOO, BNSS, and BNSeSe. Table 1 shows the calculated and experimental *k*_toRISC_ as well as *Φ*, *Φ*_Prompt_, *Φ*_TADF_, *Φ*_Phos_(T_1_), *Φ*_Phos_(T_2_), and *k*_TADF_ for BNOO, BNSS, and BNSeSe. Here, *Φ*_Prompt_, *Φ*_TADF_, *Φ*_Phos_(T_1_), and *Φ*_Phos_(T_2_) are the PLQYs of the prompt fluorescence, TADF, phosphorescence from T_1_, and phosphorescence from T_2_, respectively. *k*_TADF_ is the rate constant of TADF (delayed fluorescence). Because *Φ*_Phos_(T_1_) ≈ 0 for the three compounds, we attributed the delayed luminescence to TADF (*Φ*_Phos_(T_2_) ≈ 0 for all six compounds). Most importantly, the calculated *k*_toRISC_ increased with increasing atomic number for the chalcogen atoms, and the *k*_toRISC_ quantitatively agrees with the experimental values, which confirms the validity of our method of predicting *k*_toRISC_. The calculations here enabled us to separate the contributions of direct RISC (T_1_ → S_1_) and RISC via T_2_ (T_1_ → T_2_ → S_1_). We denote them as *k*_toRISC_(T_1_) (=*k*_RISC_(T_1_ → S_1_)) and *k*_toRISC_(T_2_), respectively. Regarding BNOO, BNSS, and BNSeSe, the rate constants for *k*_toRISC_(T_2_) are almost identical to *k*_toRISC_ (=*k*_toRISC_(T_1_) + *k*_toRISC_(T_2_)) (Table 1 and Fig. 2j–l), indicating that the T_1_ → T_2_ → S_1_ transition is the dominant RISC pathway. It should also be noted that *k*_toRISC_ is much smaller than *k*_RISC_(T_2_ → S_1_). This is because the downhill T_2_ → T_1_ IC is rapid compared with the uphill T_1_ → T_2_ IC and T_2_ → S_1_ RISC. Thus, in addition to increasing *k*_RISC_(T_2_ → S_1_), decreasing Δ*E*(T_1_ → T_2_) is also crucial for accelerating T_2_-mediated RISC.

### Computational results for BNTeTe and BNPoPo

**Fig. 3:**
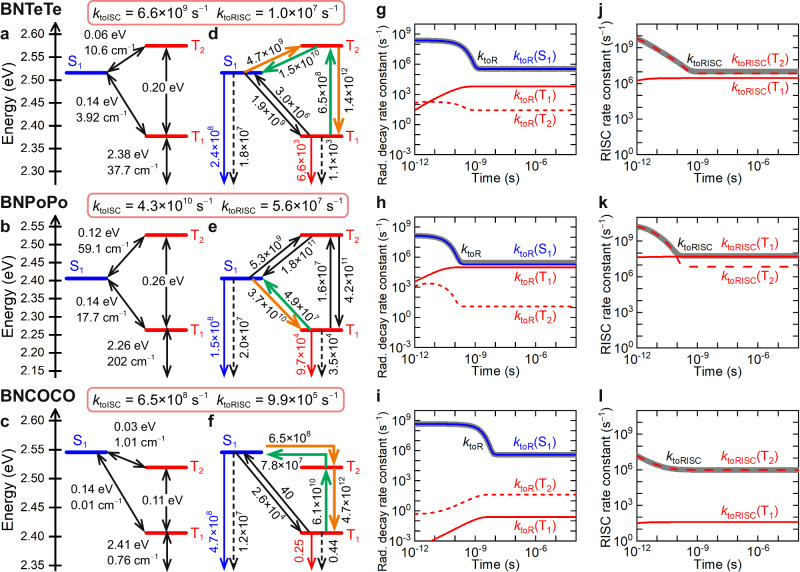
Excited-state decay mechanism. **a**–**c** Calculated excited-state energy diagram, energy differences (eV), and spin–orbit couplings (cm^−1^) and **d**–**f** rate constants (s^−1^) for BNTeTe, BNPoPo, and BNCOCO. **g**–**i** Calculated *k*_toR_, *k*_toR_(S_1_), *k*_toR_(T_1_), and *k*_toR_(T_2_) for BNTeTe, BNPoPo, and BNCOCO. **j**–**l** Calculated *k*_toRISC_, *k*_toRISC_(T_1_), and *k*_toRISC_(T_2_) for BNTeTe, BNPoPo, and BNCOCO. In **d**–**f**, the solid orange arrows depict the dominant S_1_ → T_1_ pathway, the solid green arrows depict the dominant T_1_ → S_1_ pathway, the solid black arrows depict the minor S_1_ → T_1_ and T_1_ → S_1_ pathways, the solid blue arrows depict the S_1_ → S_0_ fluorescence, the solid red arrows depict the T_1_ → S_0_ phosphorescence, and the dashed arrows show S_1_ → S_0_ and T_1_ → S_0_ nonradiative decays. In **g**–**i**, the solid grey curves depict *k*_toR_, the blue curves depict *k*_toR_(S_1_), the solid red curves depict *k*_toR_(T_1_), and the dashed red curves depict *k*_toR_(T_2_). In **j**–**l**, the solid grey curves depict *k*_toRISC_, the solid red curves depict *k*_toRISC_(T_1_), and the dashed red curves depict *k*_toRISC_(T_2_). Source data for **g**–**l** are provided as a Source Data file.

From the above discussion, we expected further enhanced RISC by further increasing the atomic number of the included atoms. Hence, we next replaced Se with Te or Po (Fig. 1d and e) to further increase the SOCs and accelerate RISC. HOMO − 1, HOMO, and LUMO of BNTeTe and BNPoPo are similar to those of BNOO, BNSS, and BNSeSe (Fig. 1). Regarding BNTeTe (Fig. 3a), the energy level alignment of S_1_, T_1_, and T_2_ is almost identical to those of BNSS and BNSeSe (Fig. 2b, c). Hence, the S/Se→Te replacement affected *k*_toRISC_ by enhancing the S_1_–T_1_ and S_1_–T_2_ SOCs. We calculated the S_0_–T_1_, S_1_–T_1_, and S_1_–T_2_ SOCs of BNTeTe to be 37.7, 3.92, and 10.6 cm^−1^, respectively, which are 3 to 4 times those of BNSeSe (13.5, 0.84, and 3.32 cm^−1^, respectively). As a result, all SOC-related rate constants (*k*_ISC_(S_1_ → T_1_), *k*_ISC_(S_1_ → T_2_), *k*_RISC_(T_2_ → S_1_), *k*_RISC_(T_1_ → S_1_), *k*_NR_(T_1_ → S_0_), and *k*_Phos_(T_1_ → S_0_)) of BNTeTe were larger than those of BNSeSe. In contrast, *k*_F_(S_1_ → S_0_) and *k*_NR_(S_1_ → S_0_) of BNTeTe were comparable to those of BNSS and BNSeSe. Thus, acceleration of RISC is possible without sacrificing radiative decay and PLQY (Fig. 3d, g, j). The contributions of direct ISC and direct RISC to total ISC and total RISC processes, respectively, were more substantial for BNTeTe than for BNSeSe. However, the stepwise T_2_-mediated process was still dominant (70% of the total process; Fig. 3j). The calculated *k*_toRISC_ of BNTeTe was on the order of 10^7^ s^−1^. *k*_Phos_ also increased but on the order of 10^3^ s^−1^; therefore, phosphorescence was still negligible (*Φ*_Phos_(T_1_) = 0.02), and T_1_ excitons were preferentially converted into light as TADF (Fig. 3g). Thus, BNTeTe is a promising MR–TADF emitter with fast RISC.

Po has a more substantial heavy atom effect than Te (Fig. 3b); hence, one can expect that BNPoPo would also be an excellent TADF emitter with much faster RISC (although Po compounds are radioactive, we performed calculations to understand the heavy atom effect). In contrast to our expectation, BNPoPo exhibited substantial phosphorescence as well as TADF (*Φ*_Phos_ = 0.28 and *Φ*_TADF_ = 0.55; Table 1 and Fig. 3e show rate constants). Therefore, BNPoPo would not be a pure TADF emitter (Fig. 3h), although it exhibited the fastest *k*_toRISC_ of 5.6 × 10^7^ s^−1^ because of the substantial heavy atom effect of Po. This is different from all the other compounds in this study (they are pure TADF emitters). Interestingly, the dominant RISC processes are found to change from T_1_ → T_2_ → S_1_ to direct T_1_ → S_1_ process at 10^-10^ s for BNPoPo (Fig. 3k). Our proposed method also provides such detailed exciton dynamics.

### Computational results for BNCOCO

We investigated another approach to accelerating RISC: C = O substitution. Figure 1f shows the designed molecule (BNCOCO). We calculated Δ*E*(T_1_ → S_1_) of BNCOCO to be 0.14 eV (Fig. 3c); which is comparable to those of BNSS, BNSeSe, BNTeTe, and BNPoPo. S_1_–T_2_ SOC of BNCOCO (1.01 cm^−1^, Fig. 3c) is only slightly smaller than that of BNSS (1.17 cm^−1^, Fig. 2b). Although T_2_ → S_1_ RISC is downhill for BNSS and uphill for BNCOCO, the energy gaps for T_2_ → S_1_ were small in both cases. Consequently, the *k*_RISC_(T_2_ → S_1_) of BNCOCO is only slightly smaller than that of BNSS. In contrast, the reduced Δ*E*(T_1_ → T_2_) induced a faster T_1_ → T_2_ transition than in BNSS, resulting in a larger *k*_toRISC_ (9.9 × 10^5^ s^−1^) than BNSS (2.5 × 10^5^ s^−1^). *k*_toISC_ and *k*_toRISC_ of BNCOCO are close to those of BNSeSe. The large spatial overlap between HOMO and LUMO + 1 around the C = O groups (Fig. 1f) lowers the T_2_ energy level, minimising Δ*E*(T_1_ → T_2_) and leading to the large *k*_toRISC_ even without chalcogen atoms.

### A method for minimising Δ*E*(T_1_ → T_2_)

Here, we would like to discuss another approach to minimise Δ*E*(T_1_ → T_2_), which is effective in increasing *k*_toRISC_. Supplementary Fig. 8a shows a case when T_1_ and T_2_ consist of HOMO → LUMO and HOMO−1 → LUMO transitions, respectively. Δ*E*(T_1_ → T_2_) can be written as Δ*E*(T_1_ → T_2_) = (*h*_HH_−*h*_H−1H−1_) + (*J*_HH_−*J*_H−1H−1_) + (*J*_HL_−*J*_H−1L_), where *h* denotes the core integral (kinetic and potential energies), *J* denotes the Coulomb integral, and *K* denotes the exchange integral^44^ (see Supplementary Method 6 for the detail). A simple approach of minimising Δ*E*(T_1_ → T_2_) is to decrease the first term *h*_HH_−*h*_H−1H−1_, which is possible by expanding the HOMO and HOMO−1 distributions (the second and third terms of Δ*E*(T_1_ → T_2_), expressed in terms of the Coulomb integrals, are not easy to control). Supplementary Fig. 8b shows a case where T_1_ and T_2_ consist of the HOMO → LUMO and HOMO → LUMO + 1 transitions, respectively. In this case, Δ*E*(T_1_ → T_2_) can be written as Δ*E*(T_1_ → T_2_) = (*h*_L+1L+1_−*h*_LL_) + (*J*_HH+1_−*J*_HL_) + (*K*_HL+1_ − *K*_HL_). For the same reason, expanding the LUMO + 1 and LUMO distributions results in an effective approach to minimise Δ*E*(T_1_ → T_2_) by decreasing *h*_L+1L+1_−*h*_LL_. Regardless of whether T_2_ is described as the HOMO−1 → LUMO or HOMO → LUMO + 1 transition, expanding molecular orbitals relevant for T_1_ and T_2_ is a simple way to decrease Δ*E*(T_1_ → T_2_) and accelerate the T_2_-mediated RISC process. Comparison of ν-DABNA-core and V-DABNA-core is a good example^45^. V-DABNA has a larger π-conjugation than ν-DABNA and hence, V-DABNA shows a smaller Δ*E*(T_1_ → T_2_) of 98 meV than ν-DABNA-core (147 meV). This trend can be also seen in several other examples^46–50^ (Supplementary Table 15).

### Exciton dynamics

The above discussion indicates that the rate constants, including those of ISC and RISC, and quantum yields can be predicted quantitatively. This method also provides quantitative details on the exciton dynamics. Figures 2g–l and 3g–l show the time evolutions of respective rate constants. Supplementary Figs. 2 and 3 also show the time evolutions of respective exciton concentrations. These analyses enable complete elucidation of the emission mechanism. As shown in Figs. 2g–l and 3g–l, the total radiative, ISC, and RISC rate constants, corresponding to experimentally observed ones, are not constant and time-dependent because they are composed of multiple processes. Initially, *k*_toR_ is very large (10^8^−10^9^ s^−1^) but decreases to 10^4^−10^5^ s^−1^. These time evolutions, corresponding to prompt and delayed emissions, respectively, are automatically calculated by our method, including the transition states. Figures 2j–l and 3j–l show that *k*_toRISC_’s are also time-dependent; initially, they are very large and stay constant after ~10^−10^−10^−9^ s. Figure 3k shows that the RISC mechanism of BNPoPo changed from the T_2_-mediated RISC to direct T_1_ → S_1_ RISC at ~0.1 ns as described above.

### Theoretically prediction of linewidths of PL spectra

Finally, we theoretically calculated and predicted the FWHM values of the emission spectra by the vertical gradient method implemented in the ORCA 5.0.3 program package^40–42^. The calculated FWHM values of BNOO, BNSS, and BNSeSe agree well with the experimental values (Table 1 and Supplementary Figs. 4–6), confirming the validity of the calculations. Regarding BNTeTe, BNPoPo, and BNCOCO, the predicted FWHM values were 47–50, 52–56, and 43–46 nm, respectively (Table 1 and Supplementary Fig. 7); which are comparable to those for BNOO, BNSS, and BNSeSe. Thus, acceleration of RISC is possible without sacrificing the FWHM of the emission spectra.

## Discussion

We comprehensively investigated the RISC mechanism of MR–TADF emitters, BNOO, BNSS, and BNSeSe by calculating all the relevant rate constants and quantum yields. The calculated values are in quantitative agreement with the experimental results. The ISC and RISC in BNOO, BNSS, and BNSeSe were found to occur predominantly via T_2_. We also found that incorporating S and Se into the molecules was effective in accelerating RISC. Therefore, BNTeTe and BNPoPo were designed to further increase *k*_RISC_. The strong heavy atom effect of Te enabled BNTeTe to exhibit *k*_RISC_ of 10^7^ s^−1^, which is larger than those of BNOO (10^4^ s^−1^), BNSS (10^5^ s^−1^), and BNSeSe (10^6^ s^−1^). Meanwhile, although BNPoPo had the largest *k*_RISC_ of 5.6 × 10^7^ s^−1^, it exhibited a substantial contribution of phosphorescence because of the excessive heavy atom effect of Po. Our findings suggest that a moderately strong SOC that does not cause phosphorescence and suitable energy alignments are the keys to accelerating RISC in pure TADF. We also investigated BNCOCO to attain large *k*_RISC_ without heavy atoms. BNCOCO had an S_1_–T_2_ SOC comparable to BNSS but a smaller T_1_–T_2_ energy difference, resulting in a larger *k*_RISC_ of 10^6^ s^−1^.

Distinct from conventional TADF, RISC has been rate-limiting in MR-TADF, and addressing this problem is currently the most important issue in further improving its characteristics. The calculated *k*_F_(S_1_ → S_0_), *Φ*, and FWHM values of the emission spectra are nearly identical for all the compounds in this study. This study reveals the solution that the rate constants of RISC can be substantially improved without sacrificing the PLQY, rate constant of radiative decay, and emission linewidth.

Finally, we would like to discuss further acceleration of RISC. The *k*_RISC_(T_2_ → S_1_) of BNTeTe was large (1.5 × 10^10^ s^−1^) because of the large SOC and downhill transition. However, the *k*_toRISC_ was three orders of magnitude smaller (1.0 × 10^7^ s^−1^). This is because in the dominant process of RISC (T_1_ → T_2_ → S_1_), *k*_IC_(T_2_ → T_1_) (1.4 × 10^12^ s^−1^) is much larger than *k*_IC_(T_1_ → T_2_) (6.5 × 10^8^ s^−1^) and *k*_RISC_(T_2_ → S_1_). The small *k*_IC_(T_1_ → T_2_) results in a slow pump-up of excitons from T_1_ to T_2_. Even if excitons are up-converted from T_1_ to T_2_, they quickly return to T_1_ ([T_2_] ≪ [T_1_]). This problem can be solved by minimising the energy difference between T_1_ and T_2_ (eventually to zero) yet preserving the T_2_ → S_1_ transition downhill. This situation corresponds to a system in which S_1_ is energetically lower than T_1_. Recently, it has been revealed that molecules with an S_1_ that is lower in energy than T_1_ can be realised despite violating Hund’s rule^51–56^. Such inverted S_1_–T_1_ (iST) systems have an ideal energy level diagram that enables the aforementioned downhill direct RISC process without the T_1_ → T_*n*_ IC process. iST molecules have frontier orbital distributions that are similar to MR-TADF; HOMO and LUMO are localised on different atoms, having short-range CT characters. Our calculation method for MR–TADF can be directly applied to designing iST molecules, enabling further enhanced RISC and a comprehensive understanding of their emission mechanisms.

In this study, we proposed a theoretical method of predicting the energy level alignments, including higher lying states as well as S_1_ and T_1,_ by combining conventional B3LYP and B2-PLYP double-hybrid functionals. Second, we devised a method of calculating RISC (and ISC) rate constants considering RISC (and ISC) mechanisms consisting of multiple pathways. Although only S_1_, T_1_, and T_2_ are involved in the emission mechanism of molecules in this study, the method proposed here is robust enough to be applied to the case where many higher-lying states are involved. The theoretical advances enable us to quantitatively predict all the relevant rate constants and quantum yields. It is demonstrated that it is important to consider the entire system for quantitative understanding of the actual emission mechanism. To quantitatively understand RISC for all the compounds here, it is not sufficient to consider only the T_1_→S_1_ process; IC plays an important role, albeit indirect. 

Our method of calculating rate constants and quantum yields is not limited to such MR-TADFs and iSTs. The range of applications is vast, including electronic transitions in, e.g. biochemical, biomedical, pharmaceutical systems, chemical reactions, and surface science. We have also confirmed the applicability of this method to a wide range of TADF emitters other than MR-type molecules and to catalytic photooxygenation to inhibit aggregation of amyloid-β peptide as a therapeutic strategy for Alzheimer’s disease^57^.

One phenomenon often consists of combinations of several elementary processes. Of these processes, only a key process has so far been focused on. However, as in the present example, all processes are often closely related. Our physics-based method is therefore important for obtaining a comprehensive understanding of phenomena and for quantitative predictions, including the time evolutions, which enable discovery of superior systems.

## Methods

### Calculations of rate constants for TADF, phosphorescence, total ISC, total RISC, and radiative decay based on excited-state populations

We calculated the rate constants for fluorescence from S_1_ to S_0_ (*k*_F_(S_1_ → S_0_)), T_*n*_ → S_0_ phosphorescence (*k*_Phos_(T_*n*_ → S_0_)), S_1_ → S_0_ nonradiative decay (*k*_NR_(S_1_ → S_0_)), T_*n*_ → S_0_ nonradiative decay (*k*_NR_(T_*n*_ → S_0_)), T_*m*_ → T_*n*_ internal conversion (*k*_IC_(T_*m*_ → T_*n*_)), S_1_ → T_*n*_ ISC (*k*_ISC_(S_1_→T_*n*_)), and T_*n*_ → S_1_ RISC (*k*_RISC_(T_*n*_ → S_1_)) (*m*, *n* = 1, 2, *m* ≠ *n*) with Supplementary Eqs. (A1)–(A18) (Supplementary Method 1). S_*n*_ (*n* ≥ 2) and T_*m*_ (*m* ≥ 3) were located 0.3 eV higher in energy than S_1_, resulting in very small contributions for all compounds in this study; therefore, we neglected their contributions to the TADF mechanism. We performed geometric optimisation and frequency analysis of S_1_ for BNOO, BNSS, BNSeSe, BNTeTe, BNPoPo, and BNCOCO by the TD-TPSSh method (Supplementary Tables 1−6 and Supplementary Fig. 1 shows the optimised geometries). Then, we performed the excited-state calculations with the TD–B3LYP method using the optimised S_1_ geometries (Supplementary Tables 7−12). For H, B, C, N, O, and S atoms, we used the 6–31G(d) basis set. For Se, Te, and Po atoms, we used the Stuttgart/Dresden pseudopotentials and basis set (SDD)^58^. The geometrical optimisations, frequency analyses, and excited-state calculations were performed with the Gaussian 16 program package (Wallingford, CT, USA)^59^.

Calculations of SOCs, vibronic coupling constants, transition dipole moments, and permanent dipole moments were carried out by the TD–B3LYP method (TD–DFT with the B3LYP functional and 6–31G(d)+SDD basis set), in which the SOCs, vibronic coupling constants, and T_1_–T_2_ transition dipole moments were calculated with the method proposed by McMurchie and Davidson^60^ (Supplementary Method 2 and Code availability below), whereas the permanent dipole moments and S_0_–S_1_ transition dipole moment were calculated with the Gaussian 16 program package. We performed the TDA–B2-PLYP calculations with the ORCA 5.0.3 program package (FACCTs, Cologne, Germany)^40–42^ and the ADC(2) and SCS–CC2 calculations with the TURBOMOLE program package^43^.

The derivations of rate constants of individual elementary processes are shown in Supplementary Method 3. Here, we show the derivations of rate constants composed of multiple processes. Previously, rate constants for prompt and delayed fluorescence and RISC have been determined from transient photoluminescence (trPL) decay curves (number of photons counted vs. time plot)^20^, which are difficult to use for separately analysing delayed fluorescence and phosphorescence, especially when their time scales are close. In this study, we propose a method of calculating these rate constants from the excited-state populations, [S_*n*_] and [T_*n*_] (*n* = 1, 2, 3, ...). Our method distinguishes delayed fluorescence and phosphorescence, which does not require calculating a trPL decay curve.

The rate for the total ISC from the excited singlet states (S_*n*_ (*n* = 1, 2, 3, ...)) to the triplet states (T_*m*_ (*m* = 1, 2, 3, ...)) can be expressed as

1 $${\sum}_{n\ge 1}\left({\sum}_{m\ge 1}{k}_{{{{{{\rm{ISC}}}}}}}({{{{{{\rm{S}}}}}}}_{n}\to {{{{{{\rm{T}}}}}}}_{m})\right)[{{{{{{\rm{S}}}}}}}_{n}],$$

which can be written in terms of the total population of the excited singlet states ${\sum}_{l\ge 1}\left[{{{{{{\rm{S}}}}}}}_{l}\right]$ as

2 $$\left\{{\sum}_{n\ge 1}\left({\sum}_{m\ge 1}{k}_{{{{{{\rm{ISC}}}}}}}({{{{{{\rm{S}}}}}}}_{n}\to {{{{{{\rm{T}}}}}}}_{m})\right)\frac{[{{{{{{\rm{S}}}}}}}_{n}]}{{\sum }_{l\ge 1}[{{{{{{\rm{S}}}}}}}_{l}]}\right\}{\sum}_{l\ge 1}[{{{{{{\rm{S}}}}}}}_{l}].$$

Therefore, the rate constant for the total ISC *k*_toISC_ can be defined as

3 $${k}_{{{{{{\rm{toISC}}}}}}}={\sum}_{n\ge 1}\left({\sum}_{m\ge 1}{k}_{{{{{{\rm{ISC}}}}}}}({{{{{{\rm{S}}}}}}}_{n}\to {{{{{{\rm{T}}}}}}}_{m})\right)\frac{[{{{{{{\rm{S}}}}}}}_{n}]}{{\sum }_{l\ge 1}[{{{{{{\rm{S}}}}}}}_{l}]}.$$

Here, the effect of *k*_IC_(S_*n*’_→S_*n*”_) on *k*_toISC_ are included in the [S_*n*_] populations because [S_*n*_] are calculated by solving the kinetic equations that include all the elementary transitions (see Supplementary Method 3).

Furthermore, the rate for the total RISC from the triplet states to the excited singlet states can be expressed as

4 $${\sum}_{m\ge 1}\left({\sum}_{n\ge 1}{k}_{{{{{{\rm{RISC}}}}}}}({{{{{{\rm{T}}}}}}}_{m}\to {{{{{{\rm{S}}}}}}}_{n})\right)[{{{{{{\rm{T}}}}}}}_{m}],$$

which can be written in terms of the total population of the triplet states ${\sum}_{l\ge 1}\left[{{{{{{\rm{T}}}}}}}_{l}\right]$ as

5 $$\left\{{\sum}_{m\ge 1}\left({\sum}_{n\ge 1}{k}_{{{{{{\rm{RISC}}}}}}}({{{{{{\rm{T}}}}}}}_{m}\to {{{{{{\rm{S}}}}}}}_{n})\right)\frac{[{{{{{{\rm{T}}}}}}}_{m}]}{{\sum }_{l\ge 1}[{{{{{{\rm{T}}}}}}}_{l}]}\right\}{\sum}_{l\ge 1}[{{{{{{\rm{T}}}}}}}_{l}].$$

Therefore, the rate constant for the total RISC *k*_toRISC_ can be defined as

6 $${k}_{{{{{{\rm{toRISC}}}}}}}={\sum}_{m\ge 1}\left({\sum}_{n\ge 1}{k}_{{{{{{\rm{RISC}}}}}}}({{{{{{\rm{T}}}}}}}_{m}\to {{{{{{\rm{S}}}}}}}_{n})\right)\frac{[{{{{{{\rm{T}}}}}}}_{m}]}{{\sum }_{l\ge 1}[{{{{{{\rm{T}}}}}}}_{l}]}.$$

Note that *k*_toISC_ and *k*_toRISC_ are functions of time (*t*) through the time dependence of [S_*n*_] and [T_*m*_], respectively. As in the case of *k*_IC_(S_*n*’_→S_*n*”_), the effect of *k*_IC_(T_*m*’_→T_*m*”_) on *k*_toRISC_ are included in the [T_*m*_] populations (see Supplementary Method 3). The values of *k*_toISC_ and *k*_toRISC_ depend on the time domain in which they are calculated. The rate for the total radiative decay from the excited singlet and triplet states is

7 $${\sum}_{n\ge 1}{k}_{{{{{{\rm{F}}}}}}}({{{{{{\rm{S}}}}}}}_{n}\to {{{{{{\rm{S}}}}}}}_{0})[{{{{{{\rm{S}}}}}}}_{n}]+{\sum}_{m\ge 1}{k}_{{{{{{\rm{Phos}}}}}}}({{{{{{\rm{T}}}}}}}_{m}\to {{{{{{\rm{S}}}}}}}_{0})[{{{{{{\rm{T}}}}}}}_{m}],$$

which can be written in terms of the total population of the excited states ${\sum}_{l\ge 1}\left[{{{{{{\rm{S}}}}}}}_{l}\right]+{\sum}_{l\ge 1}\left[{{{{{{\rm{T}}}}}}}_{l}\right]$ as

8 $$\Bigg\{{\sum}_{n\ge 1}{k}_{{{{{{\rm{F}}}}}}}({{{{{{\rm{S}}}}}}}_{n}\to 	 {{{{{{\rm{S}}}}}}}_{0})\frac{[{{{{{{\rm{S}}}}}}}_{n}]}{{\sum }_{l\ge 1}[{{{{{{\rm{S}}}}}}}_{l}]+{\sum }_{l\ge 1}[{{{{{{\rm{T}}}}}}}_{l}]} \\ 	+{\sum}_{m\ge 1}{k}_{{{{{{\rm{Phos}}}}}}}({{{{{{\rm{T}}}}}}}_{m}\to {{{{{{\rm{S}}}}}}}_{0})\frac{[{{{{{{\rm{T}}}}}}}_{m}]}{{\sum }_{l\ge 1}[{{{{{{\rm{S}}}}}}}_{l}]+{\sum }_{l\ge 1}[{{{{{{\rm{T}}}}}}}_{l}]}\Bigg\} \\ 	 \,\times \left({\sum}_{l\ge 1}[{{{{{{\rm{S}}}}}}}_{l}]+{\sum}_{l\ge 1}[{{{{{{\rm{T}}}}}}}_{l}]\right).$$

Hence, the rate constant for the total radiative decay (*k*_toR_) can be defined as

9 $${k}_{{{{{{\rm{toR}}}}}}}=	 {\sum}_{n\ge 1}{k}_{{{{{{\rm{F}}}}}}}({{{{{{\rm{S}}}}}}}_{n}\to {{{{{{\rm{S}}}}}}}_{0})\frac{[{{{{{{\rm{S}}}}}}}_{n}]}{{\sum }_{l\ge 1}[{{{{{{\rm{S}}}}}}}_{l}]+{\sum }_{l\ge 1}[{{{{{{\rm{T}}}}}}}_{l}]}\\ 	+{\sum}_{m\ge 1}{k}_{{{{{{\rm{Phos}}}}}}}({{{{{{\rm{T}}}}}}}_{m}\to {{{{{{\rm{S}}}}}}}_{0})\frac{[{{{{{{\rm{T}}}}}}}_{m}]}{{\sum }_{l\ge 1}[{{{{{{\rm{S}}}}}}}_{l}]+{\sum }_{l\ge 1}[{{{{{{\rm{T}}}}}}}_{l}]}.$$

As in *k*_toISC_ and *k*_toRISC_, *k*_toR_ is a function of *t*.

As stated previously, regarding BNOO, BNSS, BNSeSe, BNTeTe, BNPoPo, and BNCOCO, it is sufficient to consider only S_1_, T_1_, and T_2_. Hence, *k*_toISC_, *k*_toRISC_, and *k*_toR_ can be written as

10 $${k}_{{{{{{\rm{toISC}}}}}}}={k}_{{{{{{\rm{ISC}}}}}}}({{{{{{\rm{S}}}}}}}_{1}\to {{{{{{\rm{T}}}}}}}_{1})+{k}_{{{{{{\rm{ISC}}}}}}}({{{{{{\rm{S}}}}}}}_{1}\to {{{{{{\rm{T}}}}}}}_{2})$$

11 $${k}_{{{{{{\rm{toRISC}}}}}}}={k}_{{{{{{\rm{RISC}}}}}}}({{{{{{\rm{T}}}}}}}_{1}\to {{{{{{\rm{S}}}}}}}_{1})\frac{[{{{{{{\rm{T}}}}}}}_{1}]}{[{{{{{{\rm{T}}}}}}}_{1}]+[{{{{{{\rm{T}}}}}}}_{2}]}+{k}_{{{{{{\rm{RISC}}}}}}}({{{{{{\rm{T}}}}}}}_{2}\to {{{{{{\rm{S}}}}}}}_{1})\frac{[{{{{{{\rm{T}}}}}}}_{2}]}{[{{{{{{\rm{T}}}}}}}_{1}]+[{{{{{{\rm{T}}}}}}}_{2}]}$$

12 $$\begin{array}{c}{k}_{{{{{{\rm{toR}}}}}}}={k}_{{{{{{\rm{F}}}}}}}({{{{{{\rm{S}}}}}}}_{1}\to {{{{{{\rm{S}}}}}}}_{0})\frac{[{{{{{{\rm{S}}}}}}}_{1}]}{[{{{{{{\rm{S}}}}}}}_{1}]+[{{{{{{\rm{T}}}}}}}_{1}]+[{{{{{{\rm{T}}}}}}}_{2}]}+{k}_{{{{{{\rm{Phos}}}}}}}({{{{{{\rm{T}}}}}}}_{1}\to {{{{{{\rm{S}}}}}}}_{0})\frac{[{{{{{{\rm{T}}}}}}}_{1}]}{[{{{{{{\rm{S}}}}}}}_{1}]+[{{{{{{\rm{T}}}}}}}_{1}]+[{{{{{{\rm{T}}}}}}}_{2}]}\\+{k}_{{{{{{\rm{Phos}}}}}}}({{{{{{\rm{T}}}}}}}_{2}\to {{{{{{\rm{S}}}}}}}_{0})\frac{[{{{{{{\rm{T}}}}}}}_{2}]}{[{{{{{{\rm{S}}}}}}}_{1}]+[{{{{{{\rm{T}}}}}}}_{1}]+[{{{{{{\rm{T}}}}}}}_{2}]}.\end{array}$$

The contributions from T_1_ → S_1_ and T_2_ → S_1_ RISCs to *k*_toRISC_ are defined as

13 $${k}_{{{{{{\rm{toRISC}}}}}}}({{{{{{\rm{T}}}}}}}_{1})={k}_{{{{{{\rm{RISC}}}}}}}({{{{{{\rm{T}}}}}}}_{1}\to {{{{{{\rm{S}}}}}}}_{1})\frac{[{{{{{{\rm{T}}}}}}}_{1}]}{[{{{{{{\rm{T}}}}}}}_{1}]+[{{{{{{\rm{T}}}}}}}_{2}]},$$

14 $${k}_{{{{{{\rm{toRISC}}}}}}}({{{{{{\rm{T}}}}}}}_{2})={k}_{{{{{{\rm{RISC}}}}}}}({{{{{{\rm{T}}}}}}}_{2}\to {{{{{{\rm{S}}}}}}}_{1})\frac{[{{{{{{\rm{T}}}}}}}_{2}]}{[{{{{{{\rm{T}}}}}}}_{1}]+[{{{{{{\rm{T}}}}}}}_{2}]}.$$

The contributions from S_1_ → S_0_ fluorescence *k*_toR_(S_1_), T_1_ → S_0_ phosphorescence *k*_toR_(T_1_), and T_2_ → S_0_ phosphorescence *k*_toR_(T_2_) to *k*_toR_, are defined as

15 $${k}_{{{{{{\rm{toR}}}}}}}({{{{{{\rm{S}}}}}}}_{1})={k}_{{{{{{\rm{F}}}}}}}({{{{{{\rm{S}}}}}}}_{1}\to {{{{{{\rm{S}}}}}}}_{0})\frac{[{{{{{{\rm{S}}}}}}}_{1}]}{[{{{{{{\rm{S}}}}}}}_{1}]+[{{{{{{\rm{T}}}}}}}_{1}]+[{{{{{{\rm{T}}}}}}}_{2}]},$$

16 $${k}_{{{{{{\rm{toR}}}}}}}({{{{{{\rm{T}}}}}}}_{1})={k}_{{{{{{\rm{Phos}}}}}}}({{{{{{\rm{T}}}}}}}_{1}\to {{{{{{\rm{S}}}}}}}_{0})\frac{[{{{{{{\rm{T}}}}}}}_{1}]}{[{{{{{{\rm{S}}}}}}}_{1}]+[{{{{{{\rm{T}}}}}}}_{1}]+[{{{{{{\rm{T}}}}}}}_{2}]},$$

17 $${k}_{{{{{{\rm{toR}}}}}}}({{{{{{\rm{T}}}}}}}_{2})={k}_{{{{{{\rm{Phos}}}}}}}({{{{{{\rm{T}}}}}}}_{2}\to {{{{{{\rm{S}}}}}}}_{0})\frac{[{{{{{{\rm{T}}}}}}}_{2}]}{[{{{{{{\rm{S}}}}}}}_{1}]+[{{{{{{\rm{T}}}}}}}_{1}]+[{{{{{{\rm{T}}}}}}}_{2}]}.$$

Regarding fluorescent molecules with negligibly small *k*_Phos_(T_1_ → S_0_) and *k*_Phos_(T_2_ → S_0_), such as BNOO/BNSS/BNSeSe/BNTeTe/BNCOCO, *k*_toR_ ~ *k*_F_(S_1_ → S_0_) when [T_1_] ≪ [S_1_] and [T_2_]  ≪ [S_1_] (this condition holds in the time domain immediately after the S_0_ → S_1_ photoexcitation). After sufficient time has passed following the excitation, S_1_, T_1_, and T_2_ are thermally equilibrated, and [S_1_]/([S_1_] + [T_1_] +[T_2_]) becomes constant (Figs. 2 and 3, and Supplementary Figs. 2 and 3). In such a time domain, *k*_toR_ ~ *k*_F_(S_1_ → S_0_) × [S_1_]/([S_1_] + [T_1_] +[T_2_]), which can be viewed as the rate constant for TADF (*k*_toR_ ~ *k*_TADF_). For molecules that emit both fluorescence and phosphorescence, such as BNPoPo, *k*_toR_ is intrinsically the population-weighted average of *k*_F_(S_1_ → S_0_), *k*_Phos_(T_1_ → S_0_), and *k*_Phos_(T_2_ → S_0_) (Eq. (12)). The lifetimes for the total radiative decay (*τ*_toR_) and TADF (*τ*_TADF_) can be calculated as *τ*_toR_ = 1/*k*_toR_ and *τ*_TADF_ = 1/*k*_TADF_, respectively. Yersin et al. derived *τ*_toR_ for organometal complexes^61^. Equations (9) and (12) are corrected from their equations (Supplementary Method 3 shows a detailed comparison).

### Calculations of rate constants for prompt fluorescence, TADF, and RISC based on transient photoluminescence decay curves

Experimentally, TADF properties have often been discussed in terms of the RISC rate constant (*k*_toRISC_′) determined from a trPL decay curve. Here, *k*_toRISC_′ denotes the trPL-based rate constant, whilst *k*_toRISC_ denotes the excited-state population-based rate constant (Eqs. (6) or (11)). We previously proposed an expression for *k*_toRISC_′ under the assumption that the triplet states do not decay radiatively nor nonradiatively^20^:

18 $${{k}_{{{{{{\rm{toRISC}}}}}}}}^{{\prime} }=	 \frac{{k}_{{{{{{\rm{Prompt}}}}}}}+{k}_{{{{{{\rm{Delayed}}}}}}}}{2}\\ 	 \,-\sqrt{{\left(\frac{{k}_{{{{{{\rm{Prompt}}}}}}}+{k}_{{{{{{\rm{Delayed}}}}}}}}{2}\right)}^{2}-{k}_{{{{{{\rm{Prompt}}}}}}}{k}_{{{{{{\rm{Delayed}}}}}}}\left(1+\frac{{{\varPhi }}_{{{{{{\rm{Delayed}}}}}}}}{{{\varPhi }}_{{{{{{\rm{Prompt}}}}}}}}\right)},$$

where *k*_Prompt_ and *k*_Delayed_ denote the rate constants for prompt and delayed luminescence, respectively, determined by exponential fitting of a trPL decay curve. *K*_toRISC_′ cannot be applied to emitters having phosphorescent contributions such as BNPoPo. Although *k*_toRISC_ is almost identical to *k*_toRISC_′ for BNOO, BNSS, BNSeSe, BNTeTe, and BNCOCO (in which *k*_Phos_(T_1_ → S_0_) and *k*_NR_(T_1_ → S_0_) are negligibly small (Table 1)), *k*_toRISC_ is applicable to molecules that exhibit prompt fluorescence, delayed fluorescence, and phosphorescence simultaneously; which is more universal than *k*_toRISC_′.

### Supplementary information

Supplementary Information

Peer Review File

### Source data

Source Data

## Data Availability

The Gaussian 16 and ORCA input and output files are deposited in the figshare data repository [10.6084/m9.figshare]. The source data underlying Figs. 2g–l and 3g–l are provided as a Source Data file. Source data are provided with this paper.

## Code availability

The code used to generate Table 1 and Figs. 2 and 3 is available on GitHub [https://github.com/KatsuyukiShizu/d77) and Zenodo [10.5281/zenodo.11124543].
