# Supplementary material for: Quantitative prediction of rate constants and its application to organic emitters
Source: Nat Commun. 2024 Jun 3;15:4723. doi: 10.1038/s41467-024-49069-4 (PMC11148104; doi:10.1038/s41467-024-49069-4)
Supplement: Supplementary file 1 — Supplementary Information [file 41467_2024_49069_MOESM1_ESM.pdf]

Supplementary Information for

Quantitative prediction of rate constants and its application to organic emitters

*Katsuyuki Shizu[1] and Hironori Kaji[1]**

Email: kaji@scl.kyoto-u.ac.jp

[1]Institute for Chemical Research, Kyoto University, Uji, Kyoto 611-0011, Japan

Supplementary Method 1

Method of calculating rate constants

In this study, each type of rate constant was calculated using the following equations.[1,2]

1. $S_1 \rightarrow S_0$ fluorescence, $k_F(S_1 \rightarrow S_0)$

$$k_F(S_1 \rightarrow S_0) = \frac{\{E(S_1) - E(S_0)\}^3}{3\pi\varepsilon_0\hbar^4 c_3}\mu(S_1\text{-}S_0)^2 \times \frac{n_{ref}(n_{ref}{}^2 + 2)^2}{9} \quad \text{(A1)}$$

$E(S_1)$: energy level of S_1
$E(S_0)$: energy level of S_0
$\mu(S_1\text{-}S_0)$: transition electric dipole moment between S_1 and S_0
n_{ref}: refractive index of the surrounding medium (n_{ref} = 1.8)
ε_0: vacuum permittivity
$\hbar$: Dirac constant (Planck constant divided by 2π)
c: speed of light

2. $T_n \rightarrow S_0$ phosphorescence, $k_{Phos}(T_n \rightarrow S_0)$ (n, $m \geq 1$)

$$k_{Phos}(T_n \rightarrow S_0) = \frac{\{E(T_n) - E(S_0)\}^3}{3\pi\varepsilon_0\hbar^4 c_3}\mu(T_n\text{-}S_0)^2 \times \frac{n_{ref}(n_{ref}{}^2 + 2)^2}{9} \quad \text{(A2)}$$

$$\mu(T_n\text{-}S_0) = \{\mu(T_n) - \mu(S_0)\}\frac{SOC(S_0\text{-}T_n)}{E(T_n) - E(S_0)} + \sum_m \mu(S_m\text{-}S_0)\frac{SOC(S_m\text{-}T_n)}{E(T_n) - E(S_m)} + \sum_m \mu(T_n\text{-}T_m)\frac{SOC(S_0\text{-}T_m)}{E(S_0) - E(T_m)} \quad \text{(A3)}$$

$E(T_n)$: energy level of T_n
$E(T_m)$: energy level of T_m
$E(S_m)$: energy level of S_m
$\mu(T_n\text{-}S_0)$: transition electric dipole moment between T_n and S_0
$\mu(T_n)$: permanent electric dipole moment of T_n
$\mu(S_0)$: permanent electric dipole moment of S_0
$\mu(S_m\text{-}S_0)$: transition electric dipole moment of S_m
$\mu(T_n\text{-}T_m)$: transition electric dipole moment between T_n and T_m
$SOC(S_0\text{-}T_n)$: $S_0\text{-}T_n$ SOC
$SOC(S_m\text{-}T_n)$: $S_0\text{-}T_n$ SOC
$SOC(S_0\text{-}T_m)$: $S_0\text{-}T_m$ SOC

3. Energy-downhill $S_1 \rightarrow T_n$ intersystem crossing, $k_{ISC}(S_1 \rightarrow T_n)$, and energy-uphill $T_n \rightarrow S_1$ reverse intersystem crossing, $k_{RISC}(T_n \rightarrow S_1)$, where $E(S_1) > E(T_n)$

$$k_{ISC}(S_1 \rightarrow T_n) = \frac{2\pi}{\hbar}|SOC(S_1\text{-}T_n)|^2 \times LSF_{ISC}\big(E(S_1) - E(T_n)\big) \quad \text{(A4)}$$

$$\mathrm{LSF_{ISC}}\left(E(\mathrm{S_1})-E(\mathrm{T}_n)\right)=\frac{1}{\pi}\frac{\gamma}{\{E(\mathrm{S_1})-E(\mathrm{T}_n)\}^2+\gamma^2} \tag{A5}$$

$$k_{\mathrm{RISC}}(\mathrm{T}_n\rightarrow\mathrm{S_1})=\frac{1}{3}\times k_{\mathrm{ISC}}(\mathrm{S_1}\rightarrow\mathrm{T}_n)\exp[-\beta\{E(\mathrm{S_1})-E(\mathrm{T}_n)\}] \tag{A6}$$

|SOC(S_1-T_n)|: norm of the S_1-T_n SOC
γ: half width of half maximum of the line-shape function, $\mathrm{LSF_{ISC}}$
β: inverse temperature ($\beta^{-1} = k_BT$)
k_B: Boltzmann constant
T: temperature

We used FWHM = 1000 cm^{-1} (γ = 500 cm^{-1}) according to the previous report.[1]

4. Energy-uphill S_1→T_n intersystem crossing, k_{ISC}(S_1→T_n), and energy-downhill T_n→S_1 reverse intersystem crossing, k_{RISC}(T_n→S_1), where E(S_1) < E(T_n)

$$k_{\mathrm{RISC}}(\mathrm{T}_n\rightarrow\mathrm{S_1})=\frac{1}{3}\times\frac{2\pi}{\hbar}|\mathrm{SOC}(\mathrm{S_1}\text{-}\mathrm{T}_n)|^2\times\mathrm{LSF_{ISC}}\left(E(\mathrm{T}_n)-E(\mathrm{S_1})\right) \tag{A7}$$

$$k_{\mathrm{ISC}}(\mathrm{S_1}\rightarrow\mathrm{T}_n)=3k_{\mathrm{RISC}}(\mathrm{T}_n\rightarrow\mathrm{S_1})\exp[-\beta\{E(\mathrm{T}_n)-E(\mathrm{S_1})\}] \tag{A8}$$

5. S_1→S_0 nonradiative decay, k_{NR}(S_1→S_0)

$$k_{\mathrm{NR}}(\mathrm{S_1}\rightarrow\mathrm{S_0})=\sum_{\alpha}k_{\mathrm{NR},\alpha}(\mathrm{S_1}\rightarrow\mathrm{S_0}) \tag{A9}$$

$$k_{\mathrm{NR},\alpha}(\mathrm{S_1}\rightarrow\mathrm{S_0})=\frac{R_\alpha(\mathrm{S_1}\text{-}\mathrm{S_0})}{\hbar}\mathrm{LSF_{NR,\alpha}} \tag{A10}$$

$$R_\alpha(\mathrm{S_1}\text{-}\mathrm{S_0})=\frac{\hbar^2V_\alpha(\mathrm{S_1}\text{-}\mathrm{S_0})^2}{\{E(\mathrm{S_1})-E(\mathrm{S_0})\}^2} \tag{A11}$$

$$\mathrm{LSF_{NR,\alpha}}=2\pi\sinh\left(\frac{1}{2}\beta\hbar\omega_\alpha\right)\sum_{v_\alpha}\exp\left\{-\left(\frac{1}{2}+v_\alpha\right)\beta\hbar\omega_\alpha\right\}$$
$$\times\frac{1}{\pi S}\left\{\frac{v_\alpha\gamma}{\{E(\mathrm{S_1})-E(\mathrm{S_0})+\hbar\omega_\alpha\}^2+\gamma^2}+\frac{(v_\alpha+1)\gamma}{\{E(\mathrm{S_1})-E(\mathrm{S_0})-\hbar\omega_\alpha\}^2+\gamma^2}\right\} \tag{A12}$$

V_α(S_1-S_0): vibronic coupling between S_1 and S_0 for the αth vibrational mode
v_α: vibrational quantum number for the αth vibrational mode
ω_α: angular frequency for the αth vibrational mode
S: empirical parameter (= 2.1947×10^5)

6. Energy-downhill T_m→T_n internal conversion, k_{IC}(T_m→T_n), and energy-uphill T_n→T_m internal conversion, k_{IC}(T_n→T_m), where E(T_m) > E(T_n)

$$k_{\mathrm{IC}}(\mathrm{T}_m \to \mathrm{T}_n) = \sum_{\alpha} k_{\mathrm{IC},\alpha}(\mathrm{T}_m \to \mathrm{T}_n) \tag{A13}$$

$$k_{\mathrm{IC},\alpha}(\mathrm{T}_m \to \mathrm{T}_n) = \frac{R_\alpha(\mathrm{T}_m\text{-}\mathrm{T}_n)}{\hbar}\mathrm{LSF}_{\mathrm{IC},\alpha} \tag{A14}$$

$$R_\alpha(\mathrm{T}_m\text{-}\mathrm{T}_n) = \frac{\hbar^2 V_\alpha(\mathrm{T}_m\text{-}\mathrm{T}_n)^2}{\{E(\mathrm{T}_m) - E(\mathrm{T}_n)\}^2} \tag{A15}$$

$$\mathrm{LSF}_{\mathrm{IC},\alpha} = 2\pi \sinh\left(\frac{1}{2}\beta\hbar\omega_\alpha\right)\sum_{v_\alpha}\exp\left\{-\left(\frac{1}{2}+v_\alpha\right)\beta\hbar\omega_\alpha\right\}$$

$$\times\frac{1}{\pi S}\left\{\frac{v_\alpha\gamma}{\{E(\mathrm{T}_m) - E(\mathrm{T}_n) + \hbar\omega_\alpha\}^2+\gamma^2} + \frac{(v_\alpha+1)\gamma}{\{E(\mathrm{T}_m) - E(\mathrm{T}_n) - \hbar\omega_\alpha\}^2+\gamma^2}\right\} \tag{A16}$$

$$k_{\mathrm{IC}}(\mathrm{T}_n \to \mathrm{T}_m) = k_{\mathrm{IC}}(\mathrm{T}_m \to \mathrm{T}_n)\exp[-\beta\{E(\mathrm{T}_m) - E(\mathrm{T}_n)\}] \tag{A17}$$

$V_\alpha(\mathrm{T}_m\text{-}\mathrm{T}_n)$: vibronic coupling between T_m and T_n

7. $\mathrm{T}_1 \to \mathrm{S}_0$ nonradiative decay, $k_{\mathrm{NR}}(\mathrm{T}_1 \to \mathrm{S}_0)$

$$k_{\mathrm{NR}}(\mathrm{T}_1 \to \mathrm{S}_0) = \frac{2\pi}{\hbar}|\mathrm{SOC}(\mathrm{T}_1\text{-}\mathrm{S}_0)|^2 \times \frac{1}{3S}\mathrm{LSF}_{\mathrm{ISC}}\big(E(\mathrm{T}_1) - E(\mathrm{S}_0)\big) \tag{A18}$$

$|\mathrm{SOC}(\mathrm{T}_1\text{-}\mathrm{S}_0)|$: norm of the T_1-S_0 SOC

Supplementary Method 2

Method of calculating spin-orbit coupling, vibronic coupling constant, transition dipole moment, and permanent dipole moment

Spin-orbit coupling (SOC), vibronic coupling constant, and T_1-T_2 transition dipole moment were calculated using the following formulae.

1. Spin-orbit coupling between S_1 and T_n, $|\mathrm{SOC}(\mathrm{S}_1\text{-}\mathrm{T}_n)|$

$$|\mathrm{SOC}(\mathrm{S}_1\text{-}\mathrm{T}_n)| = \sqrt{\sum_{M_\mathrm{S}=0,\pm1} \left|\left\langle \Phi_{\mathrm{S}_1} \middle| \mathcal{H}_\mathrm{SOC} \middle| \Phi_{\mathrm{T}_n}^{M_\mathrm{S}} \right\rangle\right|^2} \tag{B1}$$

$$\left\langle \Phi_{\mathrm{S}_1} \middle| \mathcal{H}_\mathrm{SOC} \middle| \Phi_{\mathrm{T}_n}^{M_\mathrm{S}} \right\rangle = \frac{\mathrm{FSC}^2}{2} \sum_i \sum_A Z_A^\mathrm{eff} \left\langle \Phi_{\mathrm{S}_1} \middle| \frac{\mathbf{r}_i - \mathbf{R}_A}{|\mathbf{r}_i - \mathbf{R}_A|^3} \times \nabla \middle| \Phi_{\mathrm{T}_n}^{M_\mathrm{S}} \right\rangle \cdot \mathbf{s}_i \tag{B2}$$

Φ_{S_1}: electronic wave function of S_1 state
$\Phi_{\mathrm{T}_n}^{M_\mathrm{S}}$: electronic wave function of T_1 state
FSC: fine-structure constant
Z_A^eff : effective nuclear charge of the Ath nucleus
$\mathbf{r}_i$: cartesian coordinates of the ith electron,
$\mathbf{R}_A$: cartesian coordinates of the Ath nucleus,
$\mathbf{s}_i$: spin operator of the electron i

Φ_{S_1} and $\Phi_{\mathrm{T}_n}^{M_\mathrm{S}}$ were calculated with Gaussian 16 Rev C01 program package.[3] The integral $\left\langle \Phi_{\mathrm{S}_1} \middle| \frac{\mathbf{r}_i - \mathbf{R}_A}{|\mathbf{r}_i - \mathbf{R}_A|^3} \times \nabla \middle| \Phi_{\mathrm{T}_n}^{M_\mathrm{S}} \right\rangle$ was calculated with the method proposed by McMurchie and Davidson.[4]

2. Vibronic coupling constant between S_1 and S_0 for the αth vibrational mode, $V_\alpha(\mathrm{S}_1\text{-}\mathrm{S}_0)$

$$V_\alpha(\mathrm{S}_1\text{-}\mathrm{S}_0) = -\sum_i \sum_A Z_A \frac{\mathbf{e}_{\mathrm{MW},A}^{(\alpha)}}{\sqrt{M_A}} \cdot \left\langle \Phi_{\mathrm{S}_1} \middle| \frac{\mathbf{r}_i - \mathbf{R}_A}{|\mathbf{r}_i - \mathbf{R}_A|^3} \middle| \Phi_{\mathrm{S}_0} \right\rangle \tag{B3}$$

Z_A: nuclear charge of the Ath nucleus
M_A: mass of the Ath nucleus
$\mathbf{e}_{\mathrm{MW},A}^{(\alpha)}$: the Ath element of the eigenvector of the αth vibrational mode
Φ_{S_1}: electronic wave function of S_1
Φ_{S_0}: electronic wave function of S_0
$\mathbf{r}_i$: cartesian coordinates of the ith electron,
$\mathbf{R}_A$: cartesian coordinates of the Ath nucleus

Φ_{S_1} and Φ_{S_0} were calculated with Gaussian 16 Rev C01 program package.[3] The integral $\left\langle \Phi_{\mathrm{S}_1} \middle| \frac{\mathbf{r}_i - \mathbf{R}_A}{|\mathbf{r}_i - \mathbf{R}_A|^3} \middle| \Phi_{\mathrm{S}_0} \right\rangle$ was calculated with the method proposed by McMurchie and Davidson.[4] The formula for T_m-T_n vibronic coupling constant is obtained by replacing S_1 and S_0 with T_m and T_n, respectively.

3. T_1-T_2 transition dipole moment, $\mu(T_1\text{-}T_2)$

$$\mu(T_1\text{-}T_2) = -\langle \Phi_{T_1} | \mathbf{x} | \Phi_{T_2} \rangle \quad \text{(B4)}$$

Φ_{T_1}: electronic wave function of T_1
Φ_{T_2}: electronic wave function of T_2
x: point in the three-dimensional space

Φ_{T_1} and Φ_{T_2} were calculated with Gaussian 16 Rev C01 program package.[3] The integral $\langle \Phi_{T_1} | \mathbf{x} | \Phi_{T_2} \rangle$ was calculated with the method proposed by McMurchie and Davidson.[4]

4. S_1-S_0 transition dipole moment and permanent dipole moment of S_0, T_1, and T_2 were calculated with Gaussian 16 Rev C01 program package[3].

Supplementary Method 3

Method of calculating excited-state populations and the number of emitted photons

Transient photoluminescence decay curve is numerically calculated by the following kinetic equations for S_0, S_1, T_1, and T_2 populations. The kinetic equations were solved numerically using our own code.

$$\frac{d}{dt}[\mathrm{S_1}](t) = -\{k_{\mathrm{ISC}}(\mathrm{S_1} \to \mathrm{T_1}) + k_{\mathrm{ISC}}(\mathrm{S_1} \to \mathrm{T_2}) + k_{\mathrm{F}}(\mathrm{S_1} \to \mathrm{S_0}) + k_{\mathrm{NR}}(\mathrm{S_1} \to \mathrm{S_0})\}[\mathrm{S_1}](t)$$
$$+k_{\mathrm{RISC}}(\mathrm{T_1} \to \mathrm{S_1})[\mathrm{T_1}](t) + k_{\mathrm{RISC}}(\mathrm{T_2} \to \mathrm{S_1})[\mathrm{T_2}](t) \quad \text{(C1)}$$

$$\frac{d}{dt}[\mathrm{T_2}](t) = -\{k_{\mathrm{RISC}}(\mathrm{T_2} \to \mathrm{S_1}) + k_{\mathrm{IC}}(\mathrm{T_2} \to \mathrm{T_1}) + k_{\mathrm{Phos}}(\mathrm{T_2} \to \mathrm{S_0}) + k_{\mathrm{NR}}(\mathrm{T_2} \to \mathrm{S_0})\}[\mathrm{T_2}](t)$$
$$+k_{\mathrm{ISC}}(\mathrm{S_1} \to \mathrm{T_2})[\mathrm{S_1}](t) + k_{\mathrm{IC}}(\mathrm{T_1} \to \mathrm{T_2})[\mathrm{T_1}](t) \quad \text{(C2)}$$

$$\frac{d}{dt}[\mathrm{T_1}](t) = -\{k_{\mathrm{RISC}}(\mathrm{T_1} \to \mathrm{S_1}) + k_{\mathrm{IC}}(\mathrm{T_1} \to \mathrm{T_2}) + k_{\mathrm{Phos}}(\mathrm{T_1} \to \mathrm{S_0}) + k_{\mathrm{NR}}(\mathrm{T_1} \to \mathrm{S_0})\}[\mathrm{T_1}](t)$$
$$+k_{\mathrm{ISC}}(\mathrm{S_1} \to \mathrm{T_1})[\mathrm{S_1}](t) + k_{\mathrm{IC}}(\mathrm{T_2} \to \mathrm{T_1})[\mathrm{T_2}](t) \quad \text{(C3)}$$

$$\frac{d}{dt}[\mathrm{S_0}](t) = \{k_{\mathrm{F}}(\mathrm{S_1} \to \mathrm{S_0}) + k_{\mathrm{NR}}(\mathrm{S_1} \to \mathrm{S_0})\}[\mathrm{S_1}](t)$$
$$+\{k_{\mathrm{Phos}}(\mathrm{T_2} \to \mathrm{S_0}) + k_{\mathrm{NR}}(\mathrm{T_2} \to \mathrm{S_0})\}[\mathrm{T_2}](t)$$
$$+\{k_{\mathrm{Phos}}(\mathrm{T_1} \to \mathrm{S_0}) + k_{\mathrm{NR}}(\mathrm{T_1} \to \mathrm{S_0})\}[\mathrm{T_1}](t) \quad \text{(C4)}$$

$$N(t) = \{k_{\mathrm{F}}(\mathrm{S_1} \to \mathrm{S_0})[\mathrm{S_1}](t) + k_{\mathrm{Phos}}(\mathrm{T_1} \to \mathrm{S_0})[\mathrm{T_1}](t) + k_{\mathrm{Phos}}(\mathrm{T_2} \to \mathrm{S_0})[\mathrm{T_2}](t)\} \quad \text{(C5)}$$

$$\Phi = \int N(t)dt \quad \text{(C6)}$$

$$\Phi_{\mathrm{Phos}}(\mathrm{T_1}) = \int k_{\mathrm{Phos}}(\mathrm{T_1} \to \mathrm{S_0})[\mathrm{T_1}](t)dt \quad \text{(C7)}$$

$$\Phi_{\mathrm{Phos}}(\mathrm{T_2}) = \int k_{\mathrm{Phos}}(\mathrm{T_2} \to \mathrm{S_0})[\mathrm{T_2}](t)dt \quad \text{(C8)}$$

$[S_0(t)]$: population of S_0 at time t
$[S_1(t)]$: population of S_1 at time t
$[T_1(t)]$: population of T_1 at time t
$[T_2(t)]$: population of T_2 at time t
$k_{ISC}(S_1 \to T_1)$: rate constant for the $S_1 \to T_1$ intersystem crossing
$k_{ISC}(S_1 \to T_2)$: rate constant for the $S_1 \to T_2$ intersystem crossing
$k_F(S_1 \to S_0)$: rate constant for the $S_1 \to S_0$ radiative decay
$k_{NR}(S_1 \to S_0)$: rate constant for the $S_1 \to S_0$ nonradiative decay
$k_{RISC}(T_1 \to S_1)$: rate constant for the $T_1 \to S_1$ intersystem crossing
$k_{RISC}(T_2 \to S_1)$: rate constant for the $T_2 \to S_1$ intersystem crossing
$k_{IC}(T_2 \to T_1)$: rate constant for the $T_2 \to T_1$ internal conversion
$k_{Phos}(T_2 \to S_0)$: rate constant for the $T_2 \to S_0$ radiative decay
$k_{NR}(T_2 \to S_0)$: rate constant for the $T_2 \to S_0$ nonradiative decay
$k_{IC}(T_1 \to T_2)$: rate constant for the $T_1 \to T_2$ internal conversion
$k_{Phos}(T_1 \to S_0)$: rate constant for the $T_1 \to S_0$ radiative decay
$k_{NR}(T_1 \to S_0)$: rate constant for the $T_1 \to S_0$ nonradiative decay

$N(t)$: the number of photons emitted at time t per unit time
Φ: the total PLQY
$\Phi_{\mathrm{Phos}}(\mathrm{T_1})$: the contribution from $\mathrm{T_1} \rightarrow \mathrm{S_0}$ radiative decay toΦ
$\Phi_{\mathrm{Phos}}(\mathrm{T_2})$: the contribution from $\mathrm{T_2} \rightarrow \mathrm{S_0}$ radiative decay toΦ

In the main text, we define the lifetime for the total radiative decay as $\tau_{\mathrm{toR}} = 1/k_{\mathrm{toR}}$ and mention that τ_{toR} is a simple extension of the averaged radiative decay time proposed by Yersin et al.[3] Here, we discuss this point in detail. From Equation 9, τ_{toR} is written as

$$\tau_{\mathrm{toR}} = \frac{\sum_{l\geq 1}[\mathrm{S}_l] + \sum_{l\geq 1}[\mathrm{T}_l]}{\sum_{n\geq 1} k_{\mathrm{F}}(\mathrm{S}_n \rightarrow \mathrm{S}_0)[\mathrm{S}_n] + \sum_{m\geq 1} k_{\mathrm{Phos}}(\mathrm{T}_m \rightarrow \mathrm{S}_0)[\mathrm{T}_m]} \tag{C9}$$

The lifetimes for $\mathrm{S}_n \rightarrow \mathrm{S}_0$ fluorescence ($\tau_{\mathrm{F}}(\mathrm{S}_n \rightarrow \mathrm{S}_0)$) and $\mathrm{T}_m \rightarrow \mathrm{S}_0$ phosphorescence ($\tau_{\mathrm{Phos}}(\mathrm{T}_m \rightarrow \mathrm{S}_0)$) are written as $\tau_{\mathrm{F}}(\mathrm{S}_n \rightarrow \mathrm{S}_0) = 1/k_{\mathrm{F}}(\mathrm{S}_n \rightarrow \mathrm{S}_0)$ and $\tau_{\mathrm{Phos}}(\mathrm{T}_m \rightarrow \mathrm{S}_0) = 1/k_{\mathrm{Phos}}(\mathrm{T}_m \rightarrow \mathrm{S}_0)$, respectively ($n$, m = 1, 2, 3, …). Hence,

$$\tau_{\mathrm{toR}} = \frac{\sum_{l\geq 1}[\mathrm{S}_l] + \sum_{l\geq 1}[\mathrm{T}_l]}{\sum_{n\geq 1} \frac{1}{\tau_{\mathrm{F}}(\mathrm{S}_n \rightarrow \mathrm{S}_0)}[\mathrm{S}_n] + \sum_{m\geq 1} \frac{1}{\tau_{\mathrm{Phos}}(\mathrm{T}_m \rightarrow \mathrm{S}_0)}[\mathrm{T}_m]} \tag{C10}$$

When the excited singlet and triplet states are thermally equilibrated and $E(\mathrm{T_1}) \leq E(\mathrm{S_1})$, $[\mathrm{S}_n]$ and $[\mathrm{T}_m]$ can be written in terms of $[\mathrm{T_1}]$ and S_n-$\mathrm{T_1}$ and T_m-$\mathrm{T_1}$ energy differences

$$[\mathrm{S}_n] = \frac{1}{3}[\mathrm{T}_1]\exp\left(-\frac{E(\mathrm{S}_n) - E(\mathrm{T}_1)}{k_{\mathrm{B}}T}\right) \tag{C11}$$

$$[\mathrm{T}_m] = [\mathrm{T}_1]\exp\left(-\frac{E(\mathrm{T}_m) - E(\mathrm{T}_1)}{k_{\mathrm{B}}T}\right) \tag{C12}$$

Therefore,

$$\tau_{\mathrm{toR}} = \frac{\sum_{l\geq 1} e^{-\frac{E(\mathrm{S}_l)-E(\mathrm{T}_1)}{k_{\mathrm{B}}T}} + \sum_{l\geq 1} 3e^{-\frac{E(\mathrm{T}_l)-E(\mathrm{T}_1)}{k_{\mathrm{B}}T}}}{\sum_{n\geq 1} \frac{1}{\tau_{\mathrm{F}}(\mathrm{S}_n \rightarrow \mathrm{S}_0)} e^{-\frac{E(\mathrm{S}_n)-E(\mathrm{T}_1)}{k_{\mathrm{B}}T}} + \sum_{m\geq 1} \frac{3}{\tau_{\mathrm{Phos}}(\mathrm{T}_m \rightarrow \mathrm{S}_0)} e^{-\frac{E(\mathrm{T}_m)-E(\mathrm{T}_1)}{k_{\mathrm{B}}T}}} \tag{C13}$$

τ_{toR} is the population-weighted average of $\tau_{\mathrm{F}}(\mathrm{S}_n \rightarrow \mathrm{S}_0)$ and $\tau_{\mathrm{Phos}}(\mathrm{T}_m \rightarrow \mathrm{S}_0)$. When only $\mathrm{S_1}$ and $\mathrm{T_1}$ are considered, C13 can be written as

$$\tau_{\mathrm{toR}} = \frac{e^{-\frac{E(\mathrm{S}_1)-E(\mathrm{T}_1)}{k_{\mathrm{B}}T}} + 3}{\frac{1}{\tau_{\mathrm{F}}(\mathrm{S}_1 \rightarrow \mathrm{S}_0)} e^{-\frac{E(\mathrm{S}_1)-E(\mathrm{T}_1)}{k_{\mathrm{B}}T}} + \frac{3}{\tau_{\mathrm{Phos}}(\mathrm{T}_1 \rightarrow \mathrm{S}_0)}} \tag{C14}$$

Yersin et al. proposed the averaged radiative decay time (τ_{av}) under the assumption that $\mathrm{S_1}$ and $\mathrm{T_1}$ are thermally equilibrated

$$\tau_{\mathrm{av}} = \frac{e^{-\frac{\Delta E(\mathrm{S}_1-\mathrm{T}_1)}{k_{\mathrm{B}}T}} + 1}{\frac{1}{\tau(\mathrm{S}_1)} e^{-\frac{\Delta E(\mathrm{S}_1-\mathrm{T}_1)}{k_{\mathrm{B}}T}} + \frac{1}{\tau(\mathrm{T}_1)}} \tag{C15}$$

where $\tau(\mathrm{S_1})$ and $\tau(\mathrm{T_1})$ are the decay times for $\mathrm{S_1}$ and $\mathrm{T_1}$, respectively, and $\Delta E(\mathrm{S_1} - \mathrm{T_1})$ is the $\mathrm{S_1}$-$\mathrm{T_1}$ energy gap. Note that the factor of 3 originating from the three triplet substates does not appear explicitly in the Yersin et al.'s formula (Equation C15). Comparing Equations C14 and

C15 shows that τ_{toR} defined in this study (Equation C14) is a simple extension of the Yersin et al.'s formula (Equation C15).

When $E(S_1) < E(T_1)$ (in the case of inverted singlet-triplet excited states), $[S_n]$ and $[T_m]$ can be written in terms of $[S_1]$ and S_n-S_1 and T_m-S_1 energy differences

$$[\mathrm{S}_n] = [\mathrm{S}_1]\exp\left(-\frac{E(\mathrm{S}_n)-E(\mathrm{S}_1)}{k_\mathrm{B}T}\right) \tag{C16}$$

$$[\mathrm{T}_m] = 3[\mathrm{S}_1]\exp\left(-\frac{E(\mathrm{T}_m)-E(\mathrm{S}_1)}{k_\mathrm{B}T}\right) \tag{C17}$$

Therefore,

$$\tau_\mathrm{toR} = \frac{\sum_{l\geq 1} e^{-\frac{E(\mathrm{S}_l)-E(\mathrm{S}_1)}{k_\mathrm{B}T}} + \sum_{l\geq 1} 3e^{-\frac{E(\mathrm{T}_l)-E(\mathrm{S}_1)}{k_\mathrm{B}T}}}{\sum_{n\geq 1}\frac{1}{\tau_\mathrm{F}(\mathrm{S}_n\rightarrow\mathrm{S}_0)}e^{-\frac{E(\mathrm{S}_n)-E(\mathrm{S}_1)}{k_\mathrm{B}T}} + \sum_{m\geq 1}\frac{3}{\tau_\mathrm{Phos}(\mathrm{T}_m\rightarrow\mathrm{S}_0)}e^{-\frac{E(\mathrm{T}_m)-E(\mathrm{S}_1)}{k_\mathrm{B}T}}} \tag{C18}$$

When only S_1 and T_1 are considered, C18 can be written as

$$\tau_\mathrm{toR} = \frac{1 + 3e^{-\frac{E(\mathrm{T}_1)-E(\mathrm{S}_1)}{k_\mathrm{B}T}}}{\frac{1}{\tau_\mathrm{F}(\mathrm{S}_1\rightarrow\mathrm{S}_0)} + \frac{3}{\tau_\mathrm{Phos}(\mathrm{T}_1\rightarrow\mathrm{S}_0)}e^{-\frac{E(\mathrm{T}_1)-E(\mathrm{S}_1)}{k_\mathrm{B}T}}} \tag{C19}$$

(C19) and (C14) are essentially the same.

In the main text, we define the total ISC k_{toISC} as

$$k_\mathrm{toISC} = \sum_{n\geq 1}\left(\sum_{m\geq 1} k_\mathrm{ISC}(\mathrm{S}_n\rightarrow\mathrm{T}_m)\right)\frac{[\mathrm{S}_n]}{\sum_{l\geq 1}[\mathrm{S}_l]} \tag{C20}$$

When the excited singlet states are thermally equilibrated, from (C16) and (C20)

$$k_\mathrm{toISC} = \sum_{n\geq 1}\left(\sum_{m\geq 1} k_\mathrm{ISC}(\mathrm{S}_n\rightarrow\mathrm{T}_m)\right)\frac{e^{-\frac{E(\mathrm{S}_n)-E(\mathrm{S}_1)}{k_\mathrm{B}T}}}{\sum_{l\geq 1} e^{-\frac{E(\mathrm{S}_l)-E(\mathrm{S}_1)}{k_\mathrm{B}T}}} \tag{C21}$$

Thus, (C20) contains the effect of thermal activation and deactivation between the excited singlet states on k_{toISC}. Likewise, when the triplet states are thermally equilibrated,

$$k_\mathrm{toRISC} = \sum_{m\geq 1}\left(\sum_{n\geq 1} k_\mathrm{RISC}(\mathrm{T}_m\rightarrow\mathrm{S}_n)\right)\frac{[\mathrm{T}_m]}{\sum_{l\geq 1}[\mathrm{T}_l]} \tag{C22}$$

can be written as

$$k_\mathrm{toRISC} = \sum_{m\geq 1}\left(\sum_{n\geq 1} k_\mathrm{RISC}(\mathrm{T}_m\rightarrow\mathrm{S}_n)\right)\frac{e^{-\frac{E(\mathrm{T}_m)-E(\mathrm{T}_1)}{k_\mathrm{B}T}}}{\sum_{l\geq 1} e^{-\frac{E(\mathrm{T}_l)-E(\mathrm{T}_1)}{k_\mathrm{B}T}}} \tag{C23}$$

(C22) contains the effect of thermal activation and deactivation between the triplet states on k_{toRISC}.

Supplementary Table 1 | Nuclear coordinates of S_1 geometry of BNOO in gas phase optimised at the TD-TPSSh/6-31G(d) level of theory.

Atom	Element symbol	*x* (Å)	*y* (Å)	*z* (Å)
1	C	-0.030591	4.836110	0.422831
2	C	0.357717	6.065813	0.949661
3	C	1.290846	6.110435	1.985771
4	C	1.859043	4.922663	2.462596
5	C	1.469632	3.692895	1.937428
6	C	-1.469632	-3.692895	1.937428
7	C	-1.859043	-4.922663	2.462596
8	C	-1.290846	-6.110435	1.985771
9	C	-0.357717	-6.065813	0.949661
10	C	0.030591	-4.836110	0.422831
11	C	0.477826	3.623503	0.940309
12	C	-0.477826	-3.623503	0.940309
13	C	-0.889482	1.530142	-3.127720
14	C	-1.347847	2.766185	-3.586806
15	C	-1.363796	3.878397	-2.741981
16	C	-0.912728	3.712275	-1.429831
17	C	0.912728	-3.712275	-1.429831
18	C	1.363796	-3.878397	-2.741981
19	C	1.347847	-2.766185	-3.586806
20	C	0.889482	-1.530142	-3.127720
21	N	0.000000	-2.419601	0.394075
22	C	0.458801	-2.480292	-0.959730
23	C	0.426667	-1.331804	-1.803832
24	B	0.000000	0.000000	-1.167356
25	C	-0.426667	1.331804	-1.803832
26	C	-0.458801	2.480292	-0.959730
27	N	0.000000	2.419601	0.394075
28	C	0.000000	0.000000	3.214189
29	C	0.066567	-1.210015	2.522821
30	C	-0.003217	-1.204099	1.109402
31	C	0.000000	0.000000	0.378468
32	C	0.003217	1.204099	1.109402
33	C	-0.066567	1.210015	2.522821
34	O	-0.928886	4.837814	-0.611243
35	O	0.928886	-4.837814	-0.611243
36	H	-0.075250	6.967106	0.528318
37	H	1.593150	7.068212	2.397928
38	H	2.620928	4.953113	3.235614
39	H	1.933347	2.776733	2.286134
40	H	-1.933347	-2.776733	2.286134
41	H	-2.620928	-4.953113	3.235614
42	H	-1.593150	-7.068212	2.397928
43	H	0.075250	-6.967106	0.528318
44	H	-0.913067	0.677366	-3.799834
45	H	-1.696034	2.869136	-4.611150
46	H	-1.701320	4.856271	-3.066955

47	H	1.701320	-4.856271	-3.066955
48	H	1.696034	-2.869136	-4.611150
49	H	0.913067	-0.677366	-3.799834
50	H	0.000000	0.000000	4.299906
51	H	0.179196	-2.141367	3.066313
52	H	-0.179196	2.141367	3.066313

Supplementary Table 2 | Nuclear coordinates of S_1 geometry of BNSS in gas phase optimised at the TD-TPSSh/6-31G(d) level of theory.

Atom	Element symbol	*x* (Å)	*y* (Å)	*z* (Å)
1	C	-4.859001	-0.658323	0.010570
2	C	-6.005399	-1.130224	0.659873
3	C	-5.895403	-1.861881	1.842254
4	C	-4.633090	-2.080683	2.404577
5	C	-3.489453	-1.590501	1.778118
6	C	3.398366	-1.802436	-1.663430
7	C	4.507981	-2.363325	-2.275492
8	C	5.803840	-2.072505	-1.815485
9	C	5.974023	-1.193705	-0.752583
10	C	4.861001	-0.617440	-0.122061
11	C	-3.583093	-0.907156	0.551974
12	C	3.544378	-0.934614	-0.554248
13	C	-1.462727	3.097165	-0.990891
14	C	-2.651878	3.553801	-1.546917
15	C	-3.747434	2.693649	-1.678241
16	C	-3.626169	1.365493	-1.237089
17	C	3.667300	1.430923	1.154184
18	C	3.760018	2.751101	1.641889
19	C	2.642551	3.571274	1.558308
20	C	1.451475	3.083892	1.002957
21	N	2.428237	-0.388755	0.081826
22	C	2.476997	0.947423	0.599294
23	C	1.317321	1.774226	0.504049
24	B	-0.002314	1.140859	-0.015799
25	C	-1.302197	1.767952	-0.509910
26	C	-2.447485	0.914751	-0.634691
27	N	-2.423094	-0.420037	-0.108955
28	C	0.001383	-3.217863	0.200497
29	C	1.217762	-2.515777	0.241788
30	C	1.195619	-1.122551	0.096895
31	C	-0.004397	-0.395924	0.019933
32	C	-1.208549	-1.122182	0.011671
33	C	-1.203543	-2.538501	0.063014
34	S	-4.978848	0.236814	-1.512802
35	S	5.116382	0.424878	1.265246
36	H	-6.979645	-0.918807	0.228830
37	H	-6.789127	-2.236524	2.332639

38	H	-4.537737	-2.614668	3.345750
39	H	-2.513936	-1.732474	2.231893
40	H	2.401530	-2.005684	-2.037414
41	H	4.368765	-3.015735	-3.132168
42	H	6.670337	-2.510137	-2.301129
43	H	6.969014	-0.935695	-0.402386
44	H	-0.607952	3.766884	-0.949849
45	H	-2.731999	4.580432	-1.895798
46	H	-4.680475	3.030639	-2.118164
47	H	4.699755	3.110360	2.049138
48	H	2.695771	4.594343	1.920204
49	H	0.586091	3.738744	0.962949
50	H	0.005789	-4.301725	0.263180
51	H	2.153995	-3.048076	0.375171
52	H	-2.136325	-3.088063	-0.007886

Supplementary Table 3 | Nuclear coordinates of S_1 geometry of BNSeSe in gas phase optimised using the TD-TPSSh method. For the H, B, C, and N atoms, the 6-31G(d) basis set was used. For the Se atoms, the Stuttgart/Dresden pseudopotentials and basis set (SDD) were used.

Atom	Element symbol	*x* (Å)	*y* (Å)	*z* (Å)
1	C	0.405028	4.837447	0.756781
2	C	1.171868	5.879949	1.285342
3	C	2.338372	5.604678	1.997899
4	C	2.760589	4.277484	2.150037
5	C	2.012980	3.237532	1.611024
6	C	-2.012980	-3.237532	1.611024
7	C	-2.760589	-4.277484	2.150037
8	C	-2.338372	-5.604678	1.997899
9	C	-1.171868	-5.879949	1.285342
10	C	-0.405028	-4.837447	0.756781
11	C	0.803417	3.496113	0.928709
12	C	-0.803417	-3.496113	0.928709
13	C	-0.900012	1.514751	-3.096470
14	C	-1.403539	2.728242	-3.562394
15	C	-1.484990	3.830002	-2.709591
16	C	-1.036708	3.691136	-1.385359
17	C	1.036708	-3.691136	-1.385359
18	C	1.484990	-3.830002	-2.709591
19	C	1.403539	-2.728242	-3.562394
20	C	0.900012	-1.514751	-3.096470
21	N	-0.043534	-2.423295	0.409126
22	C	0.489382	-2.493276	-0.925481
23	C	0.427074	-1.338011	-1.773343
24	B	0.000000	0.000000	-1.148036
25	C	-0.427074	1.338011	-1.773343
26	C	-0.489382	2.493276	-0.925481

27	N	0.043534	2.423295	0.409126
28	C	0.000000	0.000000	3.225231
29	C	0.000000	-1.212354	2.532475
30	C	-0.035167	-1.200687	1.117554
31	C	0.000000	0.000000	0.392758
32	C	0.035167	1.200687	1.117554
33	C	0.000000	1.212354	2.532475
34	Se	-1.242651	5.206223	-0.180429
35	Se	1.242651	-5.206223	-0.180429
36	H	0.851538	6.906424	1.132851
37	H	2.926326	6.419773	2.409263
38	H	3.688408	4.054438	2.668532
39	H	2.358246	2.212327	1.694538
40	H	-2.358246	-2.212327	1.694538
41	H	-3.688408	-4.054438	2.668532
42	H	-2.926326	-6.419773	2.409263
43	H	-0.851538	-6.906424	1.132851
44	H	-0.892706	0.657411	-3.763249
45	H	-1.745384	2.818045	-4.590412
46	H	-1.877652	4.781246	-3.053938
47	H	1.877652	-4.781246	-3.053938
48	H	1.745384	-2.818045	-4.590412
49	H	0.892706	-0.657411	-3.763249
50	H	0.000000	0.000000	4.310809
51	H	0.036833	-2.153693	3.070726
52	H	-0.036833	2.153693	3.070726

Supplementary Table 4 | Nuclear coordinates of S_1 geometry of BNTeTe in gas phase optimised using the TD-TPSSh method. For the H, B, C, and N atoms, the 6-31G(d) basis set was used. For the Te atoms, the Stuttgart/Dresden pseudopotentials and basis set (SDD) were used.

Atom	Element symbol	*x* (Å)	*y* (Å)	*z* (Å)
1	C	-4.776822	0.826524	-0.835490
2	C	-5.696138	1.352599	-1.752086
3	C	-5.258996	1.958018	-2.929218
4	C	-3.888258	2.009073	-3.214218
5	C	-2.968384	1.474101	-2.321646
6	C	2.968384	1.474101	2.321646
7	C	3.888258	2.009073	3.214218
8	C	5.258996	1.958018	2.929218
9	C	5.696138	1.352599	1.752086
10	C	4.776822	0.826524	0.835490
11	C	-3.392862	0.893659	-1.104589
12	C	3.392862	0.893659	1.104589
13	C	-1.562406	-3.112009	0.812702
14	C	-2.798431	-3.576708	1.257576
15	C	-3.899881	-2.720693	1.288627

16	C	-3.745937	-1.394460	0.841903
17	C	3.745937	-1.394460	-0.841903
18	C	3.899881	-2.720693	-1.288627
19	C	2.798431	-3.576708	-1.257576
20	C	1.562406	-3.112009	-0.812702
21	N	2.416484	0.386903	0.209412
22	C	2.523097	-0.943189	-0.338207
23	C	1.363346	-1.790815	-0.344314
24	B	0.000000	-1.168132	0.000000
25	C	-1.363346	-1.790815	0.344314
26	C	-2.523097	-0.943189	0.338207
27	N	-2.416484	0.386903	-0.209412
28	C	0.000000	3.202138	0.000000
29	C	1.209190	2.509183	0.092723
30	C	1.195228	1.094334	0.117614
31	C	0.000000	0.371032	0.000000
32	C	-1.195228	1.094334	-0.117614
33	C	-1.209190	2.509183	-0.092723
34	Te	-5.391290	-0.063430	0.974232
35	Te	5.391290	-0.063430	-0.974232
36	H	-6.759814	1.284295	-1.542137
37	H	-5.982138	2.366354	-3.629219
38	H	-3.538141	2.444558	-4.145572
39	H	-1.908980	1.478503	-2.556374
40	H	1.908980	1.478503	2.556374
41	H	3.538141	2.444558	4.145572
42	H	5.982138	2.366354	3.629219
43	H	6.759814	1.284295	1.542137
44	H	-0.704656	-3.777405	0.851105
45	H	-2.905137	-4.603879	1.597620
46	H	-4.864617	-3.069533	1.643438
47	H	4.864617	-3.069534	-1.643438
48	H	2.905137	-4.603879	-1.597620
49	H	0.704656	-3.777405	-0.851105
50	H	0.000000	4.287743	0.000000
51	H	2.151111	3.046410	0.133236
52	H	-2.151111	3.046410	-0.133236

Supplementary Table 5 | Nuclear coordinates of S_1 geometry of BNPoPo in gas phase optimised using the TD-TPSSh method. For the H, B, C, and N atoms, the 6-31G(d) basis set was used. For the Po atoms, the Stuttgart/Dresden pseudopotentials and basis set (SDD) were used.

Atom	Element symbol	*x* (Å)	*y* (Å)	*z* (Å)
1	C	-4.782300	-0.592113	1.251140
2	C	-5.663829	-0.827989	2.312850
3	C	-5.174519	-1.073153	3.596607
4	C	-3.794939	-1.037867	3.826902

5	C	-2.916155	-0.771558	2.780102
6	C	2.668356	-1.990067	-2.058160
7	C	3.470808	-2.678496	-2.949948
8	C	4.872380	-2.563401	-2.899216
9	C	5.450151	-1.737767	-1.944454
10	C	4.646995	-1.031230	-1.033607
11	C	-3.395155	-0.567250	1.471304
12	C	3.229186	-1.138976	-1.067597
13	C	-1.552199	2.974020	-1.150174
14	C	-2.789842	3.389491	-1.625511
15	C	-3.903986	2.549203	-1.510356
16	C	-3.753379	1.299247	-0.888765
17	C	3.781130	1.440434	0.552803
18	C	3.953681	2.807135	0.856573
19	C	2.847732	3.645065	0.786687
20	C	1.596937	3.118530	0.441175
21	N	2.375742	-0.469191	-0.186097
22	C	2.542210	0.912239	0.175652
23	C	1.385923	1.761988	0.133888
24	B	-0.012524	1.121627	-0.109957
25	C	-1.360444	1.727105	-0.494789
26	C	-2.532748	0.908820	-0.330911
27	N	-2.457409	-0.321679	0.414146
28	C	-0.048022	-3.122237	0.838552
29	C	1.170404	-2.481872	0.546538
30	C	1.142797	-1.137959	0.172762
31	C	-0.041663	-0.384841	0.155097
32	C	-1.246050	-1.046985	0.458245
33	C	-1.250044	-2.422377	0.779792
34	Po	-5.449926	-0.142606	-0.815616
35	Po	5.574887	0.167615	0.541566
36	H	-6.735869	-0.821664	2.135337
37	H	-5.864756	-1.272077	4.412018
38	H	-3.402199	-1.196340	4.827600
39	H	-1.847237	-0.718853	2.962904
40	H	1.588964	-2.063372	-2.121876
41	H	3.005070	-3.297201	-3.711324
42	H	5.496720	-3.103559	-3.604177
43	H	6.531025	-1.632995	-1.904470
44	H	-0.684293	3.607283	-1.316258
45	H	-2.893164	4.356534	-2.112706
46	H	-4.869257	2.855119	-1.902351
47	H	4.932140	3.198146	1.119794
48	H	2.954744	4.704136	1.005916
49	H	0.738935	3.784119	0.416283
50	H	-0.050632	-4.177267	1.095737
51	H	2.106596	-3.030494	0.590767
52	H	-2.189707	-2.927114	0.981350

Supplementary Table 6 | Nuclear coordinates of S_1 geometry of BNCOCO in gas phase optimised at the TD-TPSSh/6-31G(d) level of theory.

Atom	Element symbol	*x* (Å)	*y* (Å)	*z* (Å)
1	C	-0.048300	4.859033	0.570623
2	C	0.359951	6.031304	1.223221
3	C	1.289809	5.988591	2.257187
4	C	1.855451	4.758720	2.624923
5	C	1.452213	3.580660	2.001920
6	C	-1.452213	-3.580660	2.001920
7	C	-1.855451	-4.758720	2.624923
8	C	-1.289809	-5.988591	2.257187
9	C	-0.359951	-6.031304	1.223221
10	C	0.048300	-4.859033	0.570623
11	C	0.455376	3.612671	1.005296
12	C	-0.455376	-3.612671	1.005296
13	C	-0.936319	1.474792	-3.104385
14	C	-1.423890	2.689509	-3.587524
15	C	-1.428731	3.812528	-2.766092
16	C	-0.934864	3.731489	-1.447809
17	C	0.934864	-3.731489	-1.447809
18	C	1.428731	-3.812528	-2.766092
19	C	1.423890	-2.689509	-3.587524
20	C	0.936319	-1.474792	-3.104385
21	N	0.000000	-2.424326	0.386795
22	C	0.468054	-2.490506	-0.960449
23	C	0.452613	-1.324365	-1.779277
24	B	0.000000	0.000000	-1.151837
25	C	-0.452613	1.324365	-1.779277
26	C	-0.468054	2.490506	-0.960449
27	N	0.000000	2.424326	0.386795
28	C	0.000000	0.000000	3.202583
29	C	0.095612	-1.208684	2.514391
30	C	0.007884	-1.210699	1.097326
31	C	0.000000	0.000000	0.375336
32	C	-0.007884	1.210699	1.097326
33	C	-0.095612	1.208684	2.514391
34	H	-0.063887	6.966108	0.869304
35	H	1.603992	6.903576	2.750507
36	H	2.630820	4.717631	3.384281
37	H	1.931195	2.641670	2.257691
38	H	-1.931195	-2.641670	2.257691
39	H	-2.630820	-4.717631	3.384281
40	H	-1.603992	-6.903576	2.750507
41	H	0.063887	-6.966108	0.869304
42	H	-0.956216	0.598692	-3.745895
43	H	-1.799744	2.758031	-4.604993
44	H	-1.796598	4.776282	-3.101753
45	H	1.796598	-4.776282	-3.101753

46	H	1.799744	-2.758031	-4.604993
47	H	0.956216	-0.598692	-3.745895
48	H	0.000000	0.000000	4.287953
49	H	0.242303	-2.133480	3.059394
50	H	-0.242303	2.133480	3.059394
51	C	0.905240	-4.949405	-0.626994
52	C	-0.905240	4.949405	-0.626994
53	O	1.457783	-6.005610	-0.963549
54	O	-1.457783	6.005610	-0.963549

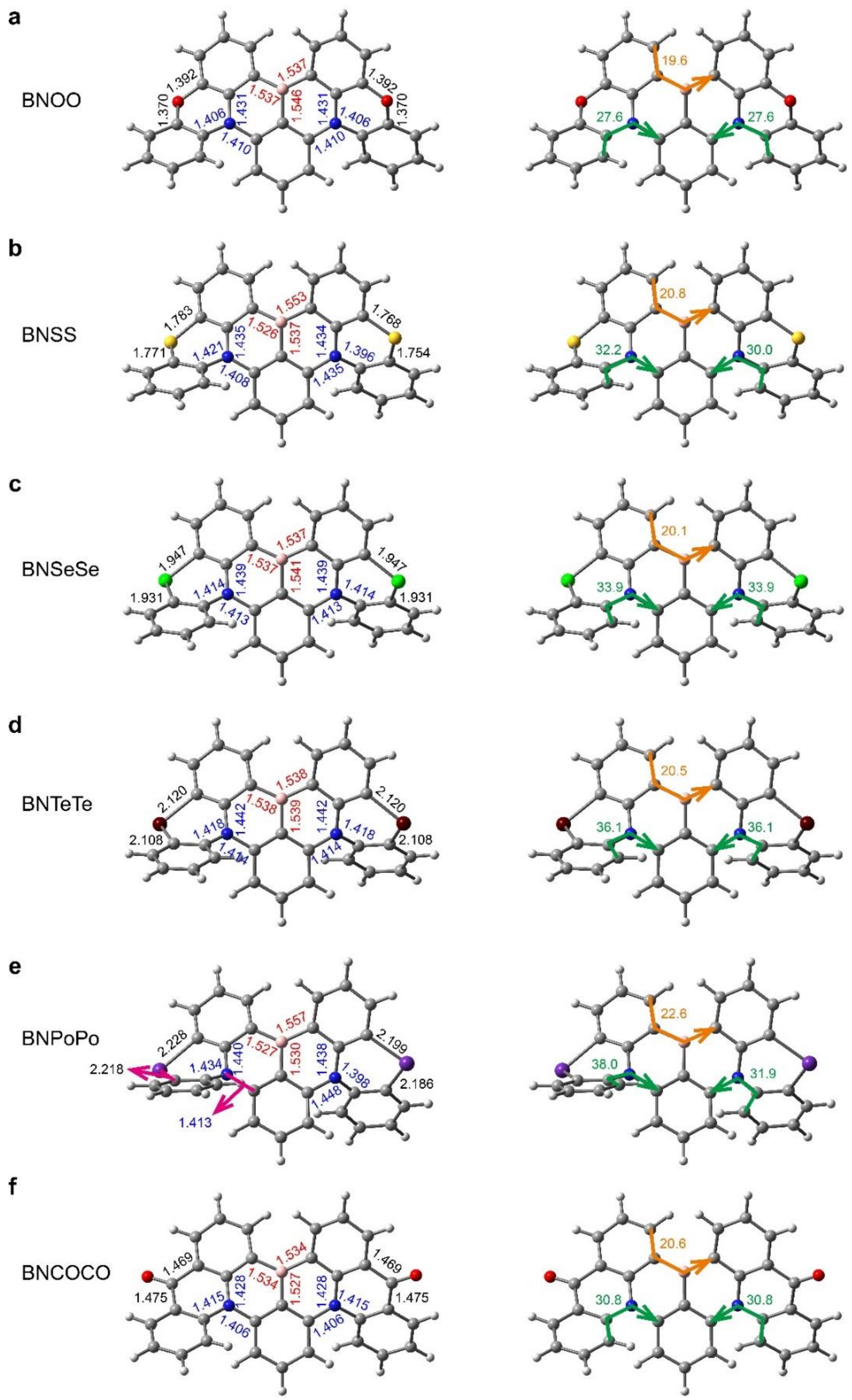

Supplementary Figure 1 | Ball-and-stick representations of the optimised S_1 geometries of (a) BNOO, (b) BNSS, (c) BNSeSe, (d) BNTeTe, (e) BNPoPo, and (f) BNCOCO. The N-C, B-C, and X-C (X = O, S, Se, Te, Po, and CO) bond lengths are in Å (left). The dihedral angles are in degree (right).

Supplementary Table 7 | S_1, S_2, T_1, T_2, and T_3 of BNOO calculated using the TD-B3LYP//TD-TPSSh method. Φ_a^r is a Slater determinant that denotes the single-electron excitation from the *a*th occupied orbital to the *r*th unoccupied orbital.

State	Excitation energy (eV)	Transition dipole moment (au)	Slater determinant	Contribution (%)
T_1	2.0902		$\Phi_{\mathrm{HOMO}}^{\mathrm{LUMO}}$	97.55
T_2	2.4251		$\Phi_{\mathrm{HOMO-1}}^{\mathrm{LUMO}}$	93.36
			$\Phi_{\mathrm{HOMO}}^{\mathrm{LUMO+5}}$	1.28
S_1	2.5052	1.702	$\Phi_{\mathrm{HOMO}}^{\mathrm{LUMO}}$	98.82
T_3	2.8443		$\Phi_{\mathrm{HOMO}}^{\mathrm{LUMO+1}}$	79.83
			$\Phi_{\mathrm{HOMO}}^{\mathrm{LUMO+7}}$	3.89
			$\Phi_{\mathrm{HOMO-3}}^{\mathrm{LUMO+7}}$	1.98
			$\Phi_{\mathrm{HOMO-1}}^{\mathrm{LUMO+2}}$	1.72
			$\Phi_{\mathrm{HOMO-2}}^{\mathrm{LUMO+1}}$	1.15
			$\Phi_{\mathrm{HOMO-5}}^{\mathrm{LUMO+1}}$	1.06
			$\Phi_{\mathrm{HOMO-4}}^{\mathrm{LUMO}}$	1.06
S_2	2.9575	0.909	$\Phi_{\mathrm{HOMO-1}}^{\mathrm{LUMO}}$	98.58

Supplementary Table 8 | S_1, S_2, T_1, T_2, and T_3 of BNSS calculated using the TD-B3LYP//TD-TPSSh method. Φ_a^r is a Slater determinant that denotes the single-electron excitation from the *a*th occupied orbital to the *r*th unoccupied orbital.

State	Excitation energy (eV)	Transition dipole moment (au)	Slater determinant	Contribution (%)
T_1	2.0879		$\Phi_{\mathrm{HOMO}}^{\mathrm{LUMO}}$	96.97
S_1	2.4388	1.607	$\Phi_{\mathrm{HOMO}}^{\mathrm{LUMO}}$	99.10
T_2	2.4742		$\Phi_{\mathrm{HOMO-1}}^{\mathrm{LUMO}}$	92.93
			$\Phi_{\mathrm{HOMO}}^{\mathrm{LUMO+5}}$	1.46
T_3	2.8686		$\Phi_{\mathrm{HOMO}}^{\mathrm{LUMO+1}}$	51.13
			$\Phi_{\mathrm{HOMO}}^{\mathrm{LUMO+3}}$	10.61
			$\Phi_{\mathrm{HOMO-2}}^{\mathrm{LUMO}}$	6.29
			$\Phi_{\mathrm{HOMO}}^{\mathrm{LUMO+2}}$	5.67
			$\Phi_{\mathrm{HOMO-1}}^{\mathrm{LUMO+3}}$	3.73
			$\Phi_{\mathrm{HOMO}}^{\mathrm{LUMO+6}}$	1.61
			$\Phi_{\mathrm{HOMO-5}}^{\mathrm{LUMO+1}}$	1.23
			$\Phi_{\mathrm{HOMO-2}}^{\mathrm{LUMO+1}}$	1.16
			$\Phi_{\mathrm{HOMO-1}}^{\mathrm{LUMO+5}}$	1.16

			$\Phi_{\mathrm{HOMO-6}}^{\mathrm{LUMO}}$	1.04
S_2	2.9046	0.892	$\Phi_{\mathrm{HOMO-1}}^{\mathrm{LUMO}}$	98.72

Supplementary Table 9 | S_1, S_2, T_1, T_2, and T_3 of BNSeSe calculated using the TD-B3LYP//TD-TPSSh method. Φ_a^r is a Slater determinant that denotes the single-electron excitation from the *a*th occupied orbital to the *r*th unoccupied orbital.

State	Excitation energy (eV)	Transition dipole moment (au)	Slater determinant	Contribution (%)
T_1	2.1630		$\Phi_{\mathrm{HOMO}}^{\mathrm{LUMO}}$	97.19
S_1	2.5105	1.653	$\Phi_{\mathrm{HOMO}}^{\mathrm{LUMO}}$	99.03
T_2	2.5557		$\Phi_{\mathrm{HOMO-1}}^{\mathrm{LUMO}}$	90.09
			$\Phi_{\mathrm{HOMO}}^{\mathrm{LUMO+5}}$	2.04
			$\Phi_{\mathrm{HOMO-3}}^{\mathrm{LUMO}}$	1.98
			$\Phi_{\mathrm{HOMO-7}}^{\mathrm{LUMO}}$	1.18
T_3	2.9395		$\Phi_{\mathrm{HOMO}}^{\mathrm{LUMO+1}}$	69.04
			$\Phi_{\mathrm{HOMO-2}}^{\mathrm{LUMO+1}}$	2.87
			$\Phi_{\mathrm{HOMO}}^{\mathrm{LUMO+5}}$	2.74
			$\Phi_{\mathrm{HOMO-4}}^{\mathrm{LUMO}}$	2.60
			$\Phi_{\mathrm{HOMO}}^{\mathrm{LUMO+7}}$	2.52
			$\Phi_{\mathrm{HOMO-1}}^{\mathrm{LUMO}}$	2.44
			$\Phi_{\mathrm{HOMO-1}}^{\mathrm{LUMO+6}}$	1.76
			$\Phi_{\mathrm{HOMO}}^{\mathrm{LUMO+11}}$	1.72
			$\Phi_{\mathrm{HOMO-3}}^{\mathrm{LUMO+6}}$	1.47
			$\Phi_{\mathrm{HOMO-8}}^{\mathrm{LUMO+11}}$	1.46
			$\Phi_{\mathrm{HOMO-5}}^{\mathrm{LUMO+1}}$	1.11
S_2	2.9543	0.498	$\Phi_{\mathrm{HOMO-1}}^{\mathrm{LUMO}}$	98.92

Supplementary Table 10 | S_1, S_2, T_1, T_2, and T_3 of BNTeTe calculated using the TD-B3LYP//TD-TPSSh method. Φ_a^r is a Slater determinant that denotes the single-electron excitation from the *a*th occupied orbital to the *r*th unoccupied orbital.

State	Excitation energy (eV)	Transition dipole moment (au)	Slater determinant	Contribution (%)
T_1	2.1827		$\Phi_{\mathrm{HOMO}}^{\mathrm{LUMO}}$	96.36
			$\Phi_{\mathrm{HOMO-2}}^{\mathrm{LUMO}}$	1.34
S_1	2.5158	1.6154	$\Phi_{\mathrm{HOMO}}^{\mathrm{LUMO}}$	99.01

T_2	2.5744		$\Phi_{\mathrm{HOMO-1}}^{\mathrm{LUMO}}$	88.03
			$\Phi_{\mathrm{HOMO-3}}^{\mathrm{LUMO}}$	4.46
			$\Phi_{\mathrm{HOMO}}^{\mathrm{LUMO+5}}$	1.84
			$\Phi_{\mathrm{HOMO-7}}^{\mathrm{LUMO}}$	1.21
T_3	2.8780		$\Phi_{\mathrm{HOMO}}^{\mathrm{LUMO+2}}$	26.51
			$\Phi_{\mathrm{HOMO-1}}^{\mathrm{LUMO+1}}$	24.82
			$\Phi_{\mathrm{HOMO-2}}^{\mathrm{LUMO}}$	23.49
			$\Phi_{\mathrm{HOMO-2}}^{\mathrm{LUMO+2}}$	8.27
			$\Phi_{\mathrm{HOMO-5}}^{\mathrm{LUMO}}$	4.27
			$\Phi_{\mathrm{HOMO-6}}^{\mathrm{LUMO}}$	1.65
S_2	2.9086	0.6065	$\Phi_{\mathrm{HOMO-1}}^{\mathrm{LUMO}}$	98.44

Supplementary Table 11 | S_1, S_2, T_1, T_2, and T_3 of BNPoPo calculated using the TD-B3LYP//TD-TPSSh method. Φ_a^r is a Slater determinant that denotes the single-electron excitation from the *a*th occupied orbital to the *r*th unoccupied orbital.

State	Excitation energy (eV)	Transition dipole moment (au)	Slater determinant	Contribution (%)
T_1	2.1339		$\Phi_{\mathrm{HOMO}}^{\mathrm{LUMO}}$	92.84
			$\Phi_{\mathrm{HOMO-2}}^{\mathrm{LUMO}}$	2.31
			$\Phi_{\mathrm{HOMO-1}}^{\mathrm{LUMO}}$	2.15
S_1	2.4062	1.3456	$\Phi_{\mathrm{HOMO}}^{\mathrm{LUMO}}$	99.23
T_2	2.5265		$\Phi_{\mathrm{HOMO-1}}^{\mathrm{LUMO}}$	84.24
			$\Phi_{\mathrm{HOMO-3}}^{\mathrm{LUMO}}$	5.28
			$\Phi_{\mathrm{HOMO-2}}^{\mathrm{LUMO+2}}$	2.90
			$\Phi_{\mathrm{HOMO}}^{\mathrm{LUMO}}$	2.15
T_3	2.6655		$\Phi_{\mathrm{HOMO}}^{\mathrm{LUMO+2}}$	81.27
			$\Phi_{\mathrm{HOMO-1}}^{\mathrm{LUMO+2}}$	9.07
			$\Phi_{\mathrm{HOMO-2}}^{\mathrm{LUMO+2}}$	4.75
S_2	2.8286	0.9483	$\Phi_{\mathrm{HOMO-1}}^{\mathrm{LUMO}}$	98.58

Supplementary Table 12 | S_1, S_2, T_1, T_2, and T_3 of BNCOCO calculated using the TD-B3LYP//TD-TPSSh method. Φ_a^r is a Slater determinant that denotes the single-electron excitation from the *a*th occupied orbital to the *r*th unoccupied orbital.

State	Excitation energy (eV)	Transition dipole moment (au)	Slater determinant	Contribution (%)

T_1	2.2005		Φ_{HOMO}^{LUMO}	95.56
			Φ_{HOMO-1}^{LUMO+1}	2.22
T_2	2.5191		Φ_{HOMO}^{LUMO+1}	51.81
			Φ_{HOMO-1}^{LUMO}	33.11
			Φ_{HOMO}^{LUMO+3}	4.78
			Φ_{HOMO-1}^{LUMO+2}	1.58
			Φ_{HOMO-5}^{LUMO+1}	1.25
S_1	2.5454	2.2007	Φ_{HOMO}^{LUMO}	98.61
T_3	2.8368		Φ_{HOMO}^{LUMO+2}	33.91
			Φ_{HOMO-1}^{LUMO+1}	26.84
			Φ_{HOMO-5}^{LUMO}	16.86
			Φ_{HOMO-3}^{LUMO}	4.17
			$\Phi_{HOMO-11}^{LUMO+1}$	1.83
			$\Phi_{HOMO-10}^{LUMO}$	1.57
			Φ_{HOMO-4}^{LUMO+1}	1.11
			Φ_{HOMO-6}^{LUMO}	1.10
			Φ_{HOMO-2}^{LUMO+1}	1.08
			Φ_{HOMO-8}^{LUMO}	1.08
S_2	3.1061	0.2341	Φ_{HOMO}^{LUMO+1}	91.50
			Φ_{HOMO-1}^{LUMO}	5.47

Supplementary Method 4

T_1-energy correction with the combined TD-B3LYP and TDA-B2-PLYP method

First, we calculated the S_1 and T_2 energies (denoted $E_{B3LYP}(S_1)$ and $E_{B3LYP}(T_2)$) using the TD-B3LYP/6-31G(d) (and SDD) method for the optimised S_1 geometry. Then, we calculated $\Delta E(T_1 \rightarrow S_1)$ (denoted as $\Delta E_{B2\text{-}PLYP}(T_1 \rightarrow S_1)$) using the TDA-B2-PLYP/def2-TZVP method for the same geometry. Finally, we calculated the T_1 energy as $E(T_1) = E_{B3LYP}(S_1) - \Delta E_{B2\text{-}PLYP}(T_1 \rightarrow S_1)$ (Table S13). For the S_1 and T_2 energies, we used the TD-B3LYP/6-31G(d) results without correction: $E(S_1) = E_{B3LYP}(S_1)$ and $E(T_2) = E_{B3LYP}(T_2)$. We used the $E(S_1)$, $E(T_1)$, and $E(T_2)$ values to calculate the rate constants.

Supplementary Table 13 | Calculated excited-state energies and energy differences with the combined TD-B3LYP and TDA-B2-PLYP method in eV. The TD-B3LYP calculations were performed with Gaussian 16 Rev C01 program package.[3] The TDA-B2-PLYP calculations were performed with ORCA 5.0.3 program package.[6-8]

	BNOO	BNSS	BNSeSe	BNTeTe	BNPoPo	BNCOCO
$E(S_1) = E_{B3LYP}(S_1)$	2.51	2.44	2.51	2.52	2.41	2.55
$E(T_2) = E_{B3LYP}(T_2)$	2.43	2.47	2.56	2.57	2.53	2.52
**$\Delta E_{B3LYP}(T_1 \rightarrow S_1)$**	**0.42**	**0.35**	**0.35**	**0.33**	**0.27**	**0.35**
**$\Delta E(T_1 \rightarrow S_1) = \Delta E_{B2\text{-}PLYP}(T_1 \rightarrow S_1)$**	**0.21**	**0.14**	**0.14**	**0.14**	**0.14**	**0.14**
$E(T_1) = E(S_1) - \Delta E(T_1 \rightarrow S_1)$	2.30	2.30	2.37	2.38	2.26	2.41
$\Delta E(T_2 \rightarrow S_1) = E(S_1) - E(T_2)$	0.08	−0.04	−0.05	−0.06	−0.12	0.03
$\Delta E(T_1 \rightarrow T_2) = E(T_2) - E(T_1)$	0.13	0.18	0.19	0.20	0.26	0.11
Experiments						
$E(S_1)$	2.54*	2.55*	2.58*			
$E(T_1)$	2.39*	2.42*	2.44*			
**$\Delta E(T_1 \rightarrow S_1)$**	**0.15***	**0.13***	**0.14***			

*) Experimental data from ref. 9.

Supplementary Table 14 | Calculated excited-state energies and energy differences with the ADC(2) and SCS-CC2 methods in eV. The def2-TZVP basis set was used for all the atoms. The ADC(2) and SCS-CC2 calculations were performed with TURBOMOLE v7.4.1 2019 program package.[10]

	BNOO	BNSS	BNSeSe	BNTeTe	BNPoPo	BNCOCO
ADC(2)						
$E_{ADC2}(S_1)$	2.84	2.30	2.35	2.35	2.33	3.07
$E_{ADC2}(T_1)$	2.61	2.22	2.27	2.27	2.25	2.83
$E_{ADC2}(T_2)$	3.14	2.67	3.23	2.77	2.71	3.03
**$\Delta E_{ADC2}(T_1 \rightarrow S_1)$**	**0.23**	**0.09**	**0.08**	**0.08**	**0.07**	**0.24**
$\Delta E_{ADC2}(T_2 \rightarrow S_1)$	-0.30	-0.37	-0.88	-0.42	-0.39	0.04
$\Delta E_{ADC2}(T_1 \rightarrow T_2)$	0.53	0.45	0.96	0.49	0.46	0.20
SCS-CC2						
$E_{SCS\text{-}CC2}(S_1)$	2.68	2.68	2.72	2.72	2.72	3.36
$E_{SCS\text{-}CC2}(T_1)$	2.58	2.58	2.63	2.64	2.64	3.09
$E_{SCS\text{-}CC2}(T_2)$	3.02	3.02	3.11	3.12	2.97	3.49
**$\Delta E_{SCS\text{-}CC2}(T_1 \rightarrow S_1)$**	**0.10**	**0.10**	**0.09**	**0.09**	**0.08**	**0.27**
$\Delta E_{SCS\text{-}CC2}(T_2 \rightarrow S_1)$	-0.34	-0.34	-0.39	-0.39	-0.25	-0.13
$\Delta E_{SCS\text{-}CC2}(T_1 \rightarrow T_2)$	0.43	0.43	0.48	0.48	0.33	0.40

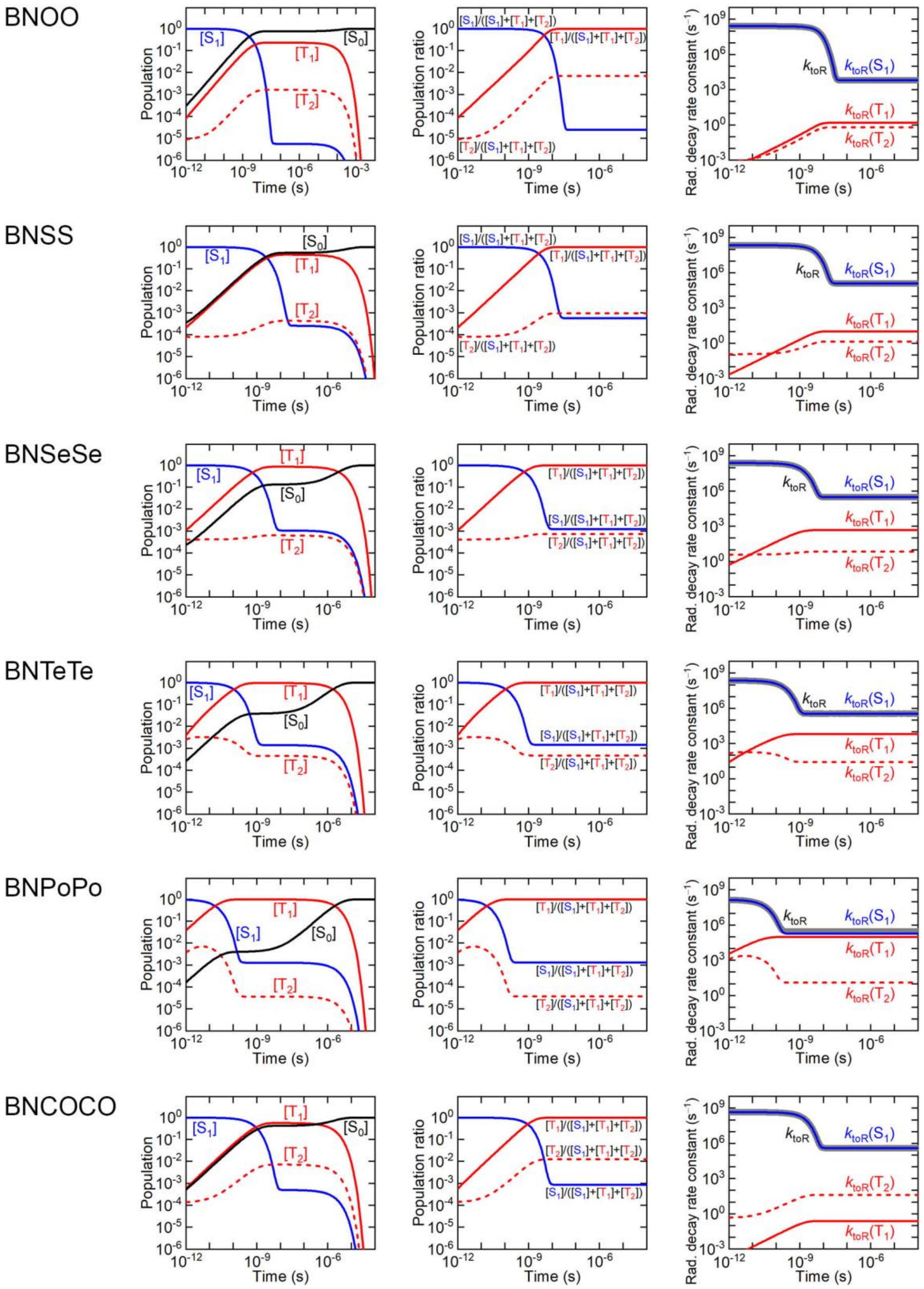

Supplementary Figure 2 | Calculated population, population ratio, and rate constants for total radiative decay for BNOO, BNSS, BNSeSe, BNTeTe, BNPoPo, and BNCOCO. $k_{toR}(S_1)$, $k_{toR}(T_1)$, and $k_{toR}(T_2)$ are the contributions from $S_1 \rightarrow S_0$ fluorescence including TADF, $T_1 \rightarrow S_0$ phosphorescence, and $T_2 \rightarrow S_0$ phosphorescence to k_{toR}, respectively: $k_{toR}=k_{toR}(S_1)+k_{toR}(T_1)+k_{toR}(T_2)$; $k_{toR}(S_1)=k_F(S_1 \rightarrow S_0)\times[S_1]/([S_1]+[T_1]+[T_2])$; $k_{toR}(T_1)=k_{Phos}(T_1S_0)\times[T_1]/([S_1]+[T_1]+[T_2])$; $k_{toR}(T_2)=k_{Phos}(T_2 \rightarrow S_0)\times[T_2]/([S_1]+[T_1]+[T_2])$. k_{toR} for BNOO/BNSS/BNSeSe/BNTeTe/BNPoPo/ BNCOCO is constant in the time domain longer than 100/100/10/10/1/10 ns.

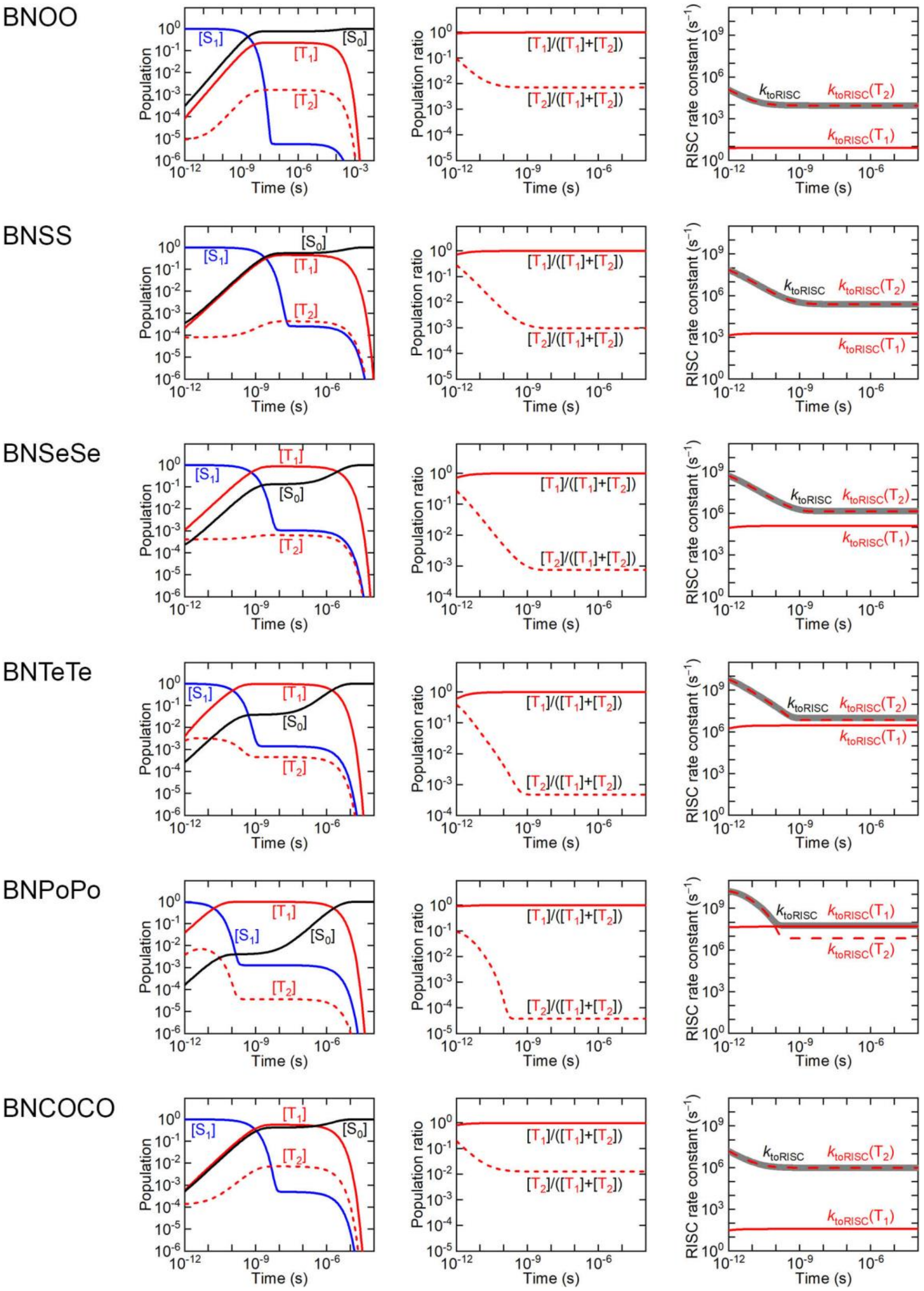

Supplementary Figure 3 | Calculated population, population ratio, and rate constants for reverse intersystem crossing (RISC) for BNOO, BNSS, BNSeSe, BNTeTe, BNPoPo, and BNCOCO. $k_{\mathrm{toRISC}}(\mathrm{T_1})$ and $k_{\mathrm{toRISC}}(\mathrm{T_2})$ are the contributions from $\mathrm{T_1 \rightarrow S_1}$ and $\mathrm{T_2 \rightarrow S_1}$ RISCs to k_{toRISC}, respectively: $k_{\mathrm{toRISC}}=k_{\mathrm{toRISC}}(\mathrm{T_1})+k_{\mathrm{toRISC}}(\mathrm{T_2})$; $k_{\mathrm{toRISC}}(\mathrm{T_1})=k_{\mathrm{RISC}}(\mathrm{T_1 \rightarrow S_1})\times[\mathrm{T_1}]/([\mathrm{T_1}]+[\mathrm{T_2}])$; $k_{\mathrm{toRISC}}(\mathrm{T_2}) = k_{\mathrm{RISC}}(\mathrm{T_2 \rightarrow S_1})\times[\mathrm{T_2}]/([\mathrm{T_1}]+[\mathrm{T_2}])$. k_{toRISC} for BNOO/BNSS/BNSeSe/BNTeTe/BNPoPo/BNCOCO is constant in the time domain longer than 1/10/10/10/1/10 ns.

Supplementary Method 5
Calculation of emission spectra

The emission spectra were calculated using the vertical gradient method implemented in ORCA 5.0.3 program package[6-8] for the S_0 geometry optimised at the PBE0/Def2-SVP level. Frequency analysis was also performed at the same level. The input file for the geometry optimisation, frequency analysis, and spectrum calculations were as follows.

```
# Geometry optimisation and frequency analysis
!PBE0 Def2-SVP Opt Freq
* XYZFILE 0 1 S0_init.xyz

# Spectrum simulation
!PBE0 DEF2-SVP TIGHTSCF ESD(FLUOR)
%TDDFT NROOTS 1
IROOT 1
END
%ESD
GSHESSIAN "S0.hess" # Use the Hessian calculated at the optimised S0-state geometry
DOHT TRUE
HESSFLAG VG # Use the vertical gradient method
LINES Gauss # Use the Gaussian distribution function
INLINEW 679.40552
SPECRES 5.0 # Spectral resolution
UNIT NM
END
* XYZFILE 0 1 S0.xyz # Use the optimised S0-state geometry
```

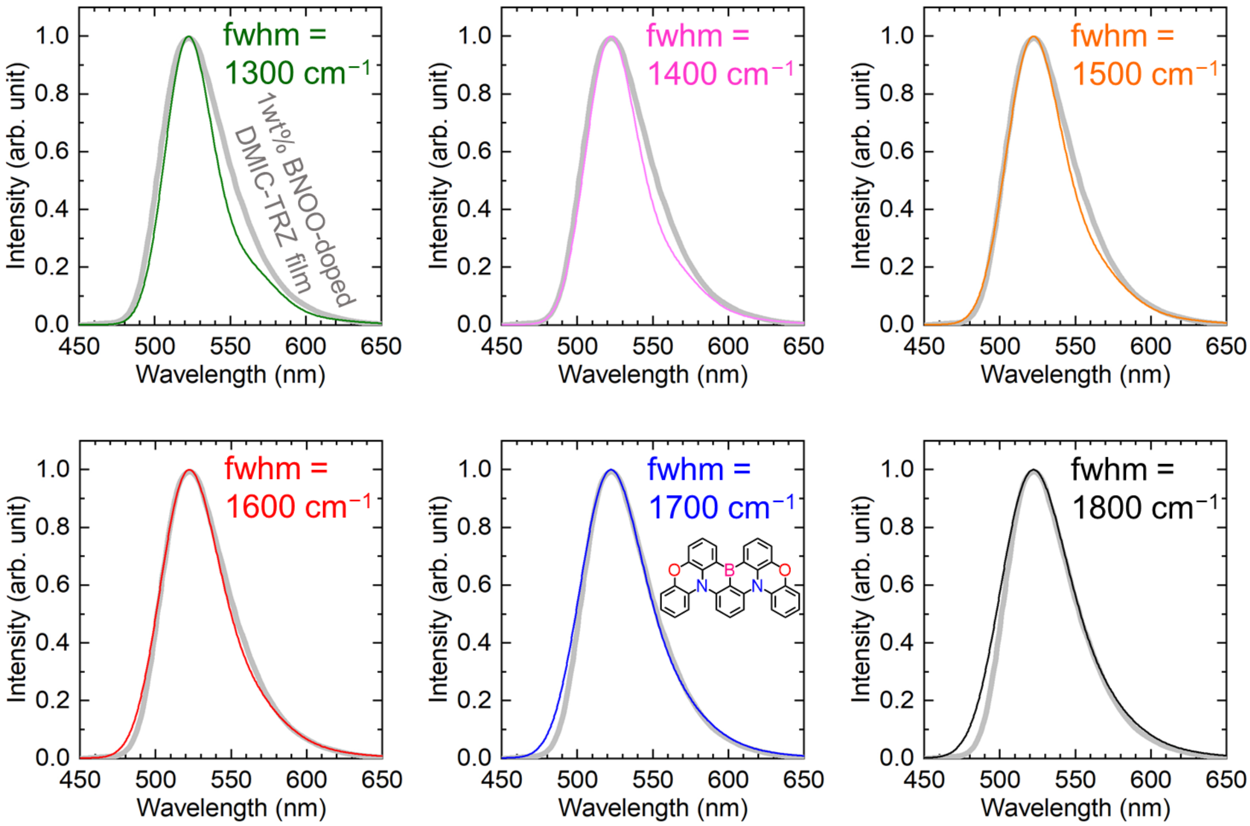

Supplementary Figure 4 | Experimental and calculated emission spectra for BNOO. The grey curve shows the experimental spectrum for 1 wt% BNOO-doped DMIC-TRZ film; the coloured curves show the calculated emission spectra. The Gaussian distribution functions with FWHM values of 1300 (green), 1400 (pink), 1500 (orange), 1600 (red), 1700 (blue), and 1800 cm^{-1} (black) were examined to reproduce the experimental spectrum. The best fit was obtained for the FWHM values of 1600 cm^{-1} (red).

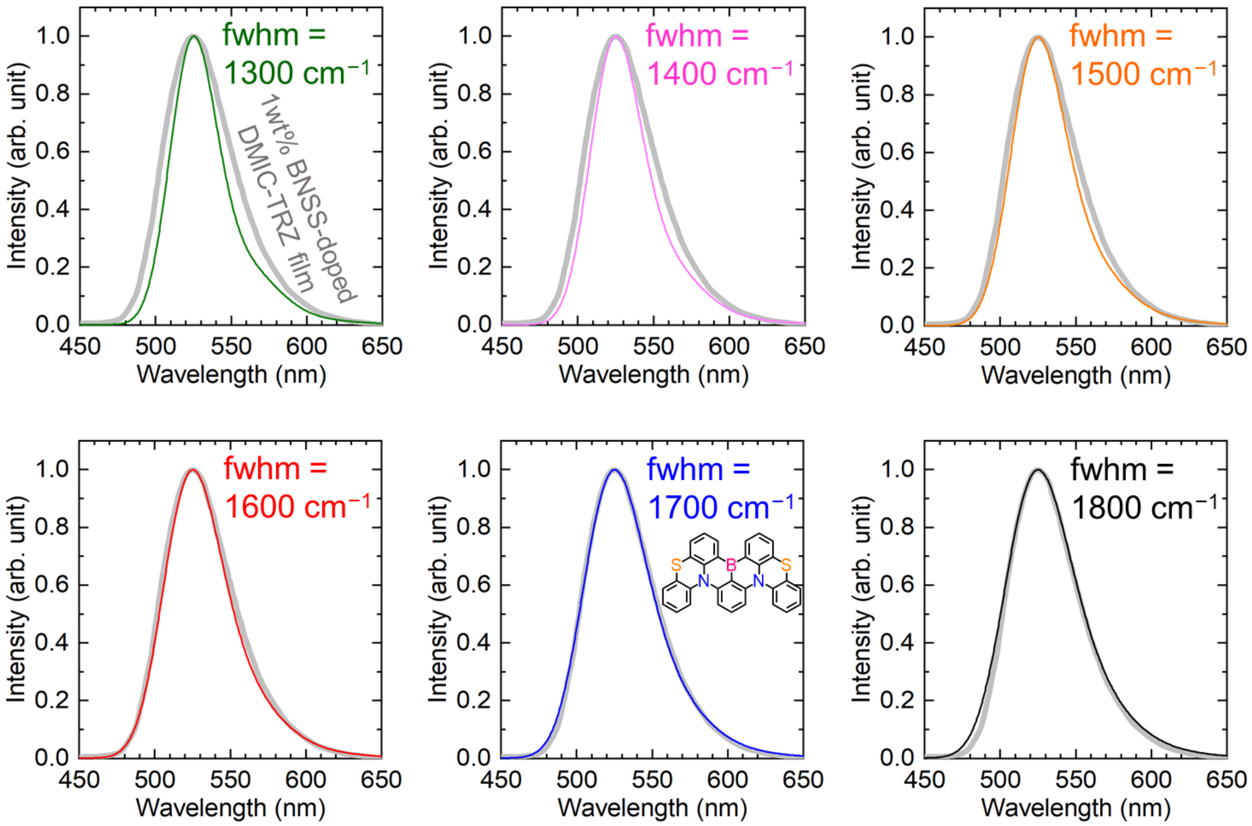

Supplementary Figure 5 | Experimental and calculated emission spectra for BNSS. The grey curve shows the experimental spectrum for 1 wt% BNSS-doped DMIC-TRZ film; the coloured curves show the calculated emission spectra. The Gaussian distribution functions with FWHM values of 1300 (green), 1400 (pink), 1500 (orange), 1600 (red), 1700 (blue), and 1800 cm^{-1} (black) were examined to reproduce the experimental spectrum. The best fit was obtained for the FWHM values of 1700 cm^{-1} (red).

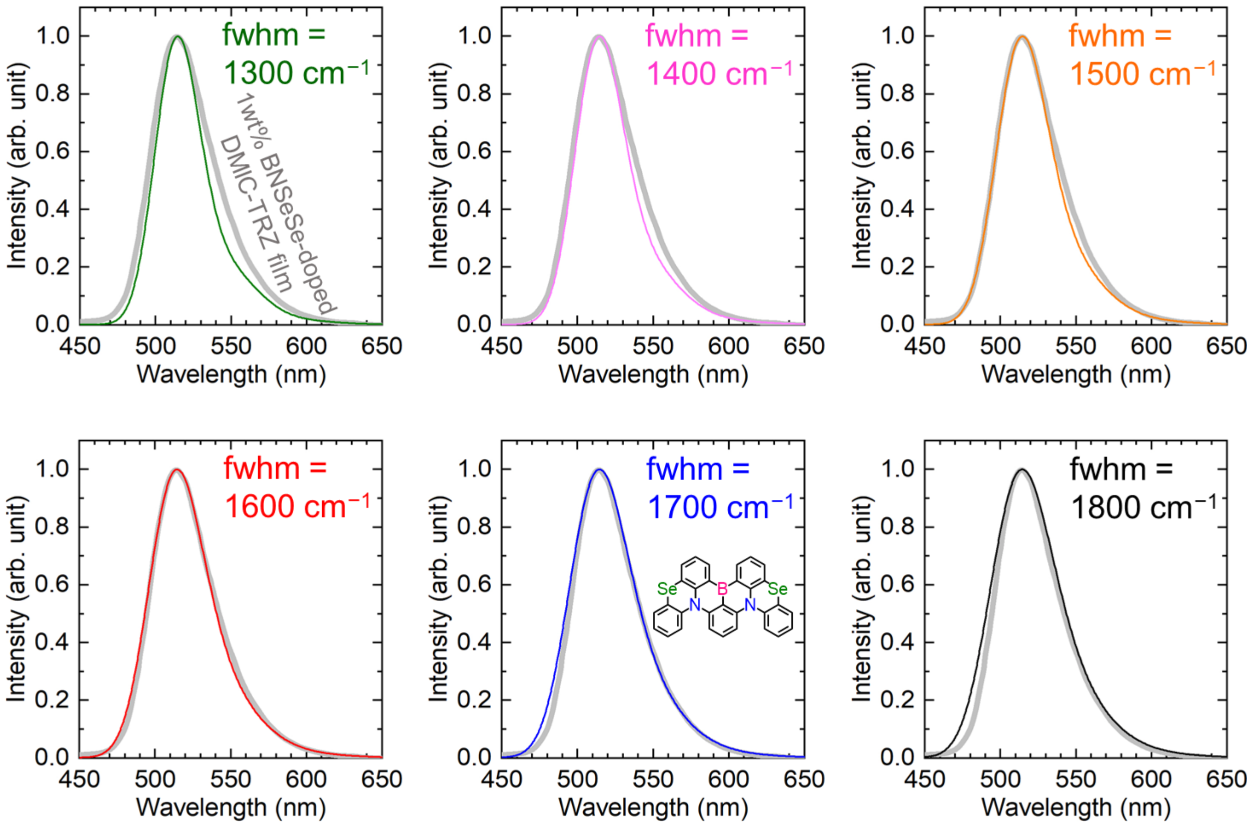

Supplementary Figure 6 | Experimental and calculated emission spectra for BNSeSe. The grey curve shows the experimental spectrum for 1 wt% BNSeSe-doped DMIC-TRZ film; the coloured curves show the calculated emission spectra. The Gaussian distribution functions with FWHM values of 1300 (green), 1400 (pink), 1500 (orange), 1600 (red), 1700 (blue), and 1800 cm^{-1} (black) were examined to reproduce the experimental spectrum. The best fit was obtained for the FWHM values of 1600 cm^{-1} (red).

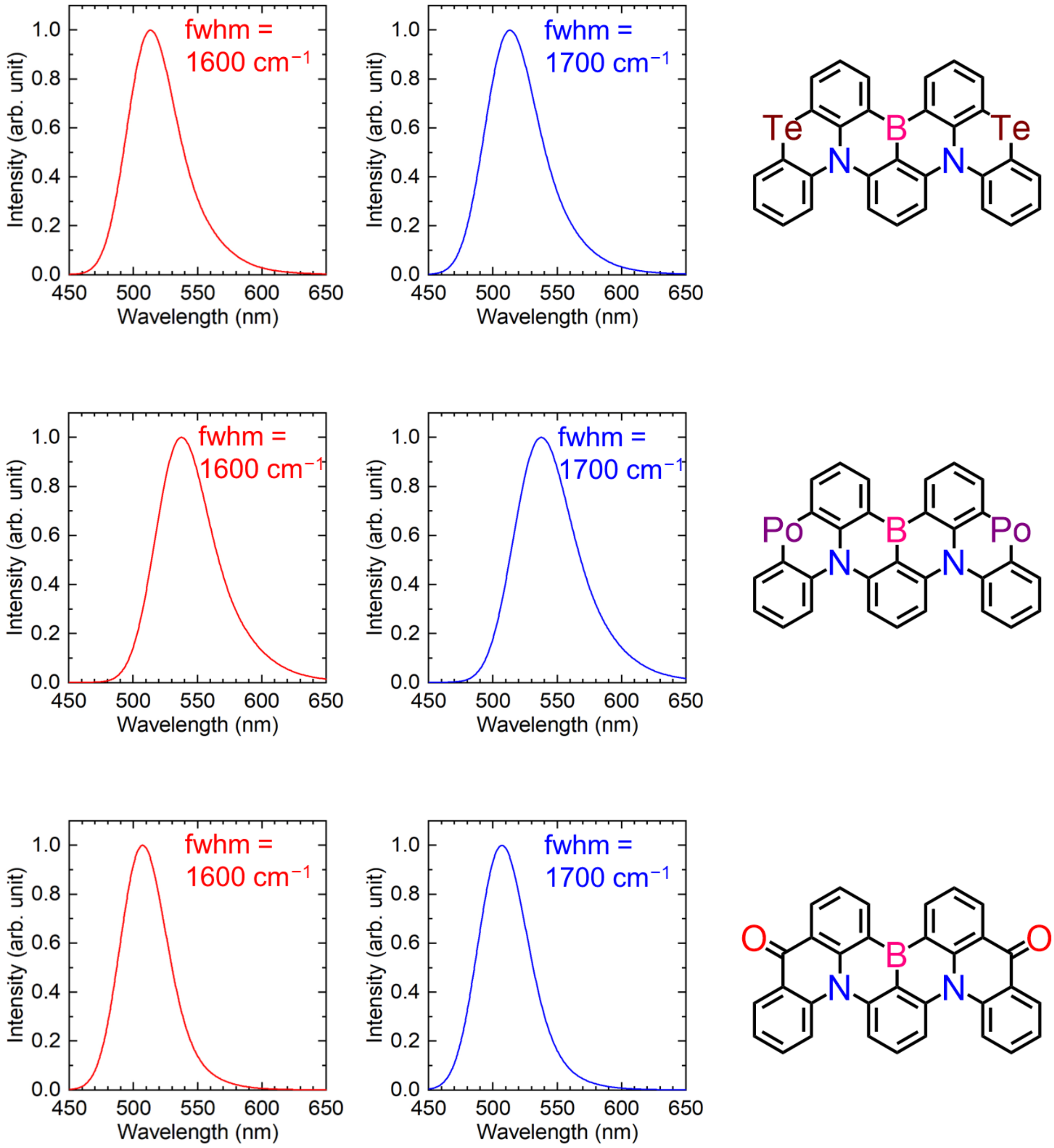

Supplementary Figure 7 | Calculated emission spectra for BNTeTe, BNPoPo, and BNCOCO. The Gaussian distribution functions with FWHM values of 1600 (red) and 1700 cm^{-1} (blue) were applied.

Supplementary Method 6
Molecular design for minimizing T_1-T_2 energy gap

Supplementary Figure 8a shows a case when T_1 and T_2 consist of HOMO → LUMO and HOMO−1 → LUMO transitions, respectively. The T_1 and T_2 energies can be expressed as[11]

$$E(\mathrm{T_1}) = 2h_{\mathrm{H-1H-1}} + h_{\mathrm{HH}} + h_{\mathrm{LL}} + J_{\mathrm{H-1H-1}} + 2J_{\mathrm{H-1H}} + 2J_{\mathrm{H-1L}} + J_{\mathrm{HL}} - K_{\mathrm{H-1H}} - K_{\mathrm{HL}} \quad \text{(D1)}$$

$$E(\mathrm{T_2}) = h_{\mathrm{H-1H-1}} + 2h_{\mathrm{HH}} + h_{\mathrm{LL}} + J_{\mathrm{HH}} + 2J_{\mathrm{H-1H}} + J_{\mathrm{H-1L}} + 2J_{\mathrm{HL}} - K_{\mathrm{H-1H}} - K_{\mathrm{HL}} \quad \text{(D2)}$$

where h denotes the core integral (kinetic and potential energies), J denotes the Coulomb integral, and K denotes the exchange integral. Then, $\Delta E(\mathrm{T_2} - \mathrm{T_1})$ is written as

$$\Delta E(\mathrm{T_2} - \mathrm{T_1}) = (h_{\mathrm{HH}} - h_{\mathrm{H-1H-1}}) + (J_{\mathrm{HH}} - J_{\mathrm{H-1H-1}}) + (J_{\mathrm{HL}} - J_{\mathrm{H-1L}}) \quad \text{(D3)}$$

A simple approach of minimizing $\Delta E(\mathrm{T_1} \rightarrow \mathrm{T_2})$ is to decrease the first term $h_{\mathrm{HH}} - h_{\mathrm{H-1H-1}}$, which is possible by expanding the HOMO and HOMO−1 distributions (the second and third terms of $\Delta E(\mathrm{T_1} \rightarrow \mathrm{T_2})$, expressed in terms of the Coulomb integrals, are not easy to control).

Supplementary Figure 8b shows a case where T_1 and T_2 consist of the HOMO → LUMO and HOMO → LUMO+1 transitions, respectively. The T_1 and T_2 energies can be expressed as

$$E(\mathrm{T_1}) = h_{\mathrm{HH}} + h_{\mathrm{LL}} + J_{\mathrm{HL}} - K_{\mathrm{HL}} \quad \text{(D4)}$$

$$E(\mathrm{T_2}) = h_{\mathrm{HH}} + h_{\mathrm{L+1L+1}} + J_{\mathrm{HL+1}} - K_{\mathrm{HL+1}} \quad \text{(D5)}$$

Hence, $\Delta E(\mathrm{T_2} - \mathrm{T_1})$ is written as

$$\Delta E(\mathrm{T_2} - \mathrm{T_1}) = (h_{\mathrm{L+1L+1}} - h_{\mathrm{LL}}) + (J_{\mathrm{HL+1}} - J_{\mathrm{HL}}) - (K_{\mathrm{HL+1}} - K_{\mathrm{HL}}) \quad \text{(D6)}$$

For the same reason, expanding the LUMO+1 and LUMO distributions results in an effective approach to minimize $\Delta E(\mathrm{T_1} \rightarrow \mathrm{T_2})$ by decreasing $h_{\mathrm{L+1L+1}} - h_{\mathrm{LL}}$. Regardless of whether T_2 is described as the HOMO−1 → LUMO or HOMO → LUMO+1 transition, expanding molecular orbitals relevant for T_1 and T_2 is a simple way to decrease $\Delta E(\mathrm{T_1} \rightarrow \mathrm{T_2})$ and accelerate the T_2-mediated RISC process.

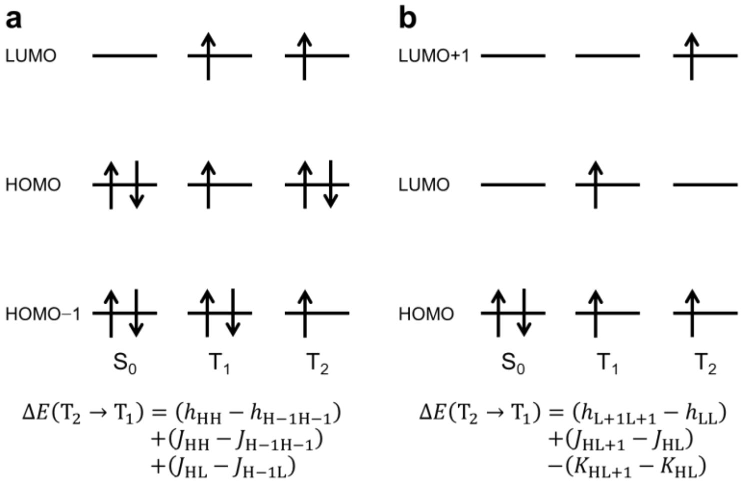

Supplementary Figure 8 | S_0, T_1, and T_2 determinants and $\Delta E(\mathrm{T_1} \rightarrow \mathrm{T_2})$. (a) A case when T_1 and T_2 consist of HOMO → LUMO (H → L) and HOMO−1 → LUMO (H−1 → L) transitions, respectively. (b) A case when T_1 and T_2 consist of HOMO → LUMO (H → L) and HOMO → LUMO+1 (H → L+1) transitions, respectively.

Supplementary Table 15 | Minimization of Δ*E*(T_1→T_2). These examples demonstrate that expanding molecular orbitals relevant for T_1 and T_2 is an effective way to decrease Δ*E*(T_1→ T_2), resulting in acceleration of the T_2-mediated RISC process.

MR-TADF molecules with Δ*E*(T_2–T_1)	References (in the main text)
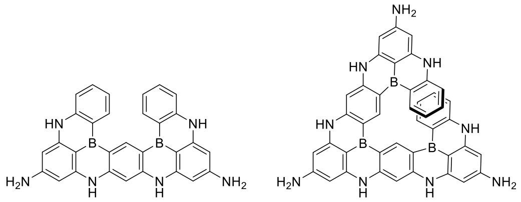 0.147 eV > 0.098 eV	12 (45)
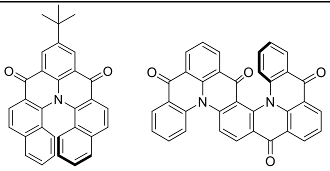 0.28 eV > 0.12 eV	13 (46)
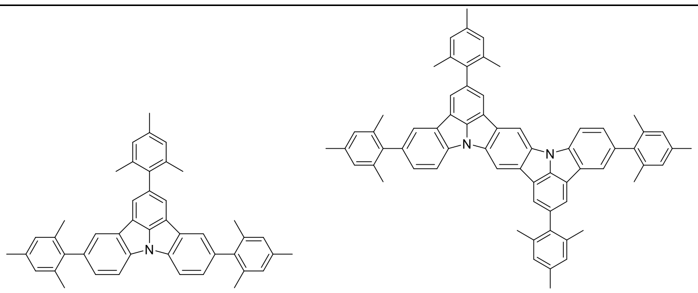 0.46 eV > 0.33 eV	14 (47)
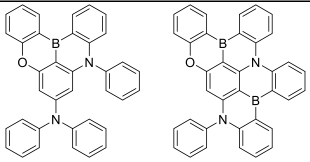 0.21 eV > 0.07 eV	15 (48)
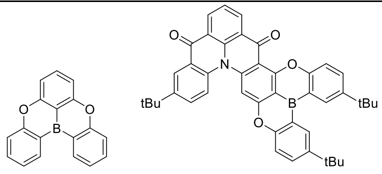 0.58 eV > 0.28 eV	16 (49)
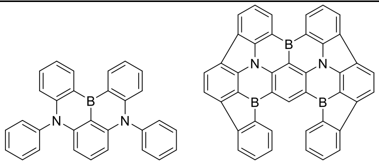 0.81 eV > 0.04 eV	17 (50)

Supplementary References

1. Shizu, K. & Kaji, H. Theoretical determination of rate constants from excited states: application to benzophenone. *J. Phys. Chem. A* **125**, 9000-9010 (2021).
2. Shizu, K. & Kaji, H. Comprehensive understanding of multiple resonance thermally activated delayed fluorescence through quantum chemistry calculations. *Commun. Chem.* **5**, 53 (2022).
3. Frisch, M. J. *et al.* Gaussian 16 Rev. C.01. (2016).
4. McMurchie, L. E. & Davidson, E. R. One- and two-electron integrals over cartesian gaussian functions. *J. Comput. Phys.* **26**, 218-231 (1978).
5. Yersin, H., Rausch, A. F., Czerwieniec, R., Hofbeck, T. & Fischer, T. The triplet state of organo-transition metal compounds. Triplet harvesting and singlet harvesting for efficient OLEDs. *Coord. Chem. Rev.* **255**, 2622-2652 (2011).
6. Neese, F. The ORCA program system. *Wiley Interdiscip. Rev.: Comput. Mol. Sci.* **2**, 73-78 (2012).
7. Neese, F. Software update: the ORCA program system, version 4.0. *WIREs Comput. Mol. Sci.* **8**, e1327 (2017).
8. Neese, F., Wennmohs, F., Becker, U. & Riplinger, C. The ORCA quantum chemistry program package. *J. Chem. Phys.* **152**, 224108 (2020).
9. Hu, Y. X., Miao, J., Hua, T., Huang, Z., Qi, Y., Zou, Y., Qiu, Y., Xia, H., Liu, H., Cao, X. & Yang, C. Efficient selenium-integrated TADF OLEDs with reduced roll-off. *Nat. Photon.* **16**, 803–810 (2022)
10. TURBOMOLE V7.4.1 2019, a development of University of Karlsruhe and Forschungszentrum Karlsruhe GmbH, 1989–2007, TURBOMOLE GmbH, since 2007.
11. Szabo, A. & Ostlund, N. S. *Modern quantum chemistry: Introduction to advanced electronic structure theory* 87-89 (Dover Publications, New York, 1996).
12. Oda, S. *et al.* One-shot synthesis of expanded heterohelicene exhibiting narrowband thermally activated delayed fluorescence. *J. Am. Chem. Soc.* **144**, 106-112 (2022).
13. Dos Santos, J. M. et al. An s-shaped double helicene showing both multi-resonance thermally activated delayed fluorescence and circularly polarized luminescence. *J. Mater. Chem. C* **10**, 4861-4870 (2022).
14. Hall, D. et al. Diindolocarbazole – achieving multiresonant thermally activated delayed fluorescence without the need for acceptor units. *Mater. Horiz.* **9**, 1068-1080 (2022).
15. Jin, J. et al. Integrating asymmetric O−B−N unit in multi-resonance thermally activated delayed fluorescence emitters towards high-performance deep-blue organic light-emitting diodes. *Angew. Chem. Int. Ed.* **62**, e202218947 (2023).
16. Wu, S. et al. Merging boron and carbonyl based mr-tadf emitter designs to achieve high performance pure blue oleds. *Angew. Chem. Int. Ed.* **62**, e202305182 (2023).
17. Keruckiene, R. et al. Is a small singlet–triplet energy gap a guarantee of TADF performance in MR-TADF compounds? Impact of the triplet manifold energy splitting. *J. Mater. Chem. C* **12**, 3450-3464 (2024).
